# Preparedness, impacts, and responses of public health emergencies towards health security: qualitative synthesis of evidence

**DOI:** 10.1186/s13690-023-01223-y

**Published:** 2023-11-30

**Authors:** Resham B Khatri, Aklilu Endalamaw, Daniel Erku, Eskinder Wolka, Frehiwot Nigatu, Anteneh Zewdie, Yibeltal Assefa

**Affiliations:** 1 Health Social Science and Development Research Institute, Kathmandu, Nepal; 2 School of Public Health, University of Queensland, Brisbane, Australia; 3 College of Medicine and Health Sciences, Bahir Dar University, Bahir Dar, Ethiopia; 4 Centre for Applied Health Economics, School of Medicine, Griffith University, Brisbane, Australia; 5 Menzies Health Institute Queensland, Griffith University, Brisbane, Australia; 6 International Institute for Primary Health Care-Ethiopia, Addis Ababa, Ethiopia

**Keywords:** Impacts, Responses, Preparedness, Public health emergencies, Primary health care, Primary care, Health security and universal health coverage

## Abstract

**Background:**

Natural and human-made public health emergencies (PHEs), such as armed conflicts, floods, and disease outbreaks, influence health systems including interruption of delivery and utilization of health services, and increased health service needs. However, the intensity and types of impacts of these PHEs vary across countries due to several associated factors. This scoping review aimed to synthesise available evidence on PHEs, their preparedness, impacts, and responses.

**Methods:**

We conducted a scoping review of published evidence. Studies were identified using search terms related to two concepts: health security and primary health care. We used Preferred Reporting Items for Systematic Reviews and Meta-Analyses extension for scoping reviews (PRISMA-ScR) guidelines to select studies. We adapted the review framework of Arksey and O’Malley. Data were analyzed using a thematic analysis approach and explained under three stages of PHEs: preparedness, impacts, and responses.

**Results:**

A total of 64 studies were included in this review. Health systems of many low- and middle-income countries had inadequate preparedness to absorb the shocks of PHEs, limited surveillance, and monitoring of risks. Health systems have been overburdened with interrupted health services, increased need for health services, poor health resilience, and health inequities. Strategies of response to the impact of PHEs included integrated services such as public health and primary care, communication and partnership across sectors, use of digital tools, multisectoral coordination and actions, system approach to responses, multidisciplinary providers, and planning for resilient health systems.

**Conclusions:**

Public health emergencies have high impacts in countries with weak health systems, inadequate preparedness, and inadequate surveillance mechanisms. Better health system preparedness is required to absorb the impact, respond to the consequences, and adapt for future PHEs. Some potential response strategies could be ensuring need-based health services, monitoring and surveillance of post-emergency outbreaks, and multisectoral actions to engage sectors to address the collateral impacts of PHEs. Mitigation strategies for future PHEs could include risk assessment, disaster preparedness, and setting digital alarm systems for monitoring and surveillance.

**Supplementary Information:**

The online version contains supplementary material available at 10.1186/s13690-023-01223-y.

**Table:** 

**Text box 1.** Contributions to the literature
• There is limited evidence synthesis on preparedness, impacts, and response of public health emergencies towards health security and universal access to health services in public health emergency settings.
• Public health emergencies have health and collateral impacts in countries with weak health systems, inadequate preparedness, and inadequate monitoring and surveillance mechanisms.
• Developing and strengthening of resilience health system is needed with strong preparedness, provision of service primary health care services, monitoring and surveillance of post-emergency outbreaks.
• The role of non-health sector and multisectoral policy and actions are crucial to address the collateral impacts of public health emergencies.

## Introduction

Health security protects against health threats by preventing, detecting, and responding to public health emergencies (PHEs) that arise from catastrophic health events or acute shocks. Such catastrophic events can be human-made (e.g., armed conflicts, forced migrations, and pandemics) or natural disasters caused by biological, geophysical, and climatological hazards, and environmental (e.g., impacts of climate change) [1–3]. Several underlying factors, including political unrest, instability leading to conflicts, and unmanaged displaced populations, contribute to the scale and complexity of PHEs. In addition, environmental degradation associated with climate change/global warming triggers new and re-emerging diseases and can contribute to drug-resistance pathogens [4]. Natural disasters can damage public health ecosystems, such as water and sanitation and waste management systems, resulting in increased health service needs and overburdened health systems [5, 6].

Public health emergencies can directly impact all six health system building blocks (services delivery, medical commodities, health workforce, governance, information system, and financing) [7]. The direct consequences of PHEs include interruption of access to and delivery of health services [8]. In contrast, collateral impacts of PHEs on non-health sectors include damage to road networks and infrastructure, shortage of food and other essential materials, and interruption of supply chain systems [9]. Priority populations – women, children, those living with disabilities, the elderly, and those with low socioeconomic status – can be highly exposed and vulnerable to the impact of events, that could lead to further marginalisation [10]. For example, the recent COVID-19 pandemic impacted these priority populations directly (e.g., increased infections) and indirectly (e.g., lockdown effects, job loss or reduced working hours) [11].

The primary health care (PHC) approach is the most suitable for early response to PHEs. The PHC approach incorporates multisectoral policy and actions and emphasises human dignity and rights [12]. Community-based PHC systems could provide comprehensive, affordable, and acceptable health services at the first point of contact in the PHE contexts [13, 14]. Responses to PHEs can be ensured by developing interdisciplinary teams, designing comprehensive interventions, and working with civil societies and communities [15, 16]. Ensuring health system preparedness, including onset and alert, is vital to mitigate the impacts of PHEs [17]. Moreover, reviewing and synthesising lessons learned from past events has been essential in responding to future PHEs. This scoping review aimed to synthesise available evidence on impacts and lessons learned of response in PHEs. The findings could inform stakeholders to identify potential strategies for responding and mitigating the PHEs consequences and building resilience in health systems.

## Methods

### Study design

We conducted a scoping review of published evidence reporting health security and primary health care utilisation in PHEs. We followed Preferred Reporting Items for Systematic Reviews and Meta-Analyses extension for scoping reviews (PRISMA-ScR) guidelines to select studies [18] (See supplementary information, Table S1). In addition, we followed the methodological framework of Arksey and O’Malley (2005) which was further refined by Levac and colleagues (2010) [19]. The resulting framework comprised of the following five steps: (a) identifying research questions, (b) identifying relevant studies, (c) selection of studies, (d) extraction and charting of data and (e) summarising and reporting results.

### Identifying research question

The following questions guided the scoping review: (1) How are health systems prepared to respond PHEs? (2) What impact did PHEs have on health care systems and services? (3) How did health systems respond to PHEs, and what are the lessons learned? To effectively answer these questions, we adopted the population, concept and context framework developed by the JBI (2015) [20] [Table 1].

**Table 1:** Population concept context (PCC) framework for defining the eligibility criteria

Criteria	Elements	Description
P- population	All people Health workforces	All individuals are accessing health care services. Health care workers such as physicians, nurses, midwives, and paramedics work as frontline contact in the health care system, and displaced populations.
C- concept	Public health emergencies	Natural disasters- earthquakes, floods. Outbreaks- epidemics and pandemics of diseases. Armed conflicts- forced migrations, political unrest.
C-context	Health services and systems • Preparedness • Impacts • Response	The ability and readiness of the health system to avail materials and human resources to provide general and essential services in PHE contexts. The impacts of PHEs have on the provision of uptake and access to health services. The capacity of the health systems to mobilise the required resources and response quickly to address the adverse consequences of PHEs.

### Identifying relevant studies

We searched eight electronic databases (PubMed, Scopus, EMBASE, CINAHL, Cochrane, Web of Science, PsycINFO, and Google Scholar) and grey literature for studies describing health security and PHC. This was followed by complementary reference searches of included studies and google searches to identify eligible studies that were not picked from the databases. The keywords used in the search strategy were built on two key concepts and tailored to each database: Health security (health security, epidemics, pandemics, outbreaks, disasters, conflictS, emergencies), and Primary Health Care. Boolean operators (AND/OR) and truncations varied depending on the databases included in the search. We included articles published in English up to 30 October 2022, but no country-related limitations were applied.

### Selection of studies

We included all relevant studies (e.g., quantitative, qualitative, mixed methods, review, reports, and further analysis of secondary data) covering health security and PHC. Data were managed using EndNote 20. The screening was undertaken based on the title and abstract initially by the first author and further assessed by the second author. This was followed by a full-text screening initially by the first author and assessed by the third author. Any disagreements were resolved by discussion. A study was included in the review if the data contributed to our research question rather than the quality of individual study. Studies were included based on the findings and their interpretation rather than as inclusion criteria itself [21, 22].

### Data charting process

A data extraction sheet was developed covering author, year, country, types of study, types of PHEs, main concepts, and key findings related to a research question (See supplementary information, Table S2). Data were extracted by the first author and double-checked by the second author. Any disagreements were resolved by discussion.

### Summarizing and reporting the results

We used Braun and Clarke’s (2006) inductive thematic analysis approach [23]. We adapted the data analysis framework proposed by Thomas et al., (2020), which denotes resilience at different stages of the PHEs cycle: health system preparedness, shock onset and alert, shock impact and management, and recovery and learning [24]. For this scoping review, we modified it into three stages: preparedness, impacts of PHEs, and responses to impacts, including impact management, recovery, and learning [11].

### Patient and public involvement

The scoping review did not involve patient populations or the general public. Their input was not sought in the scoping review design, interpretation of results, or drafting or editing of this paper. This study used secondary data; thus, ethical approval by an institutional review board was not needed.

## Results

### Description of studies

**Fig. 1:**
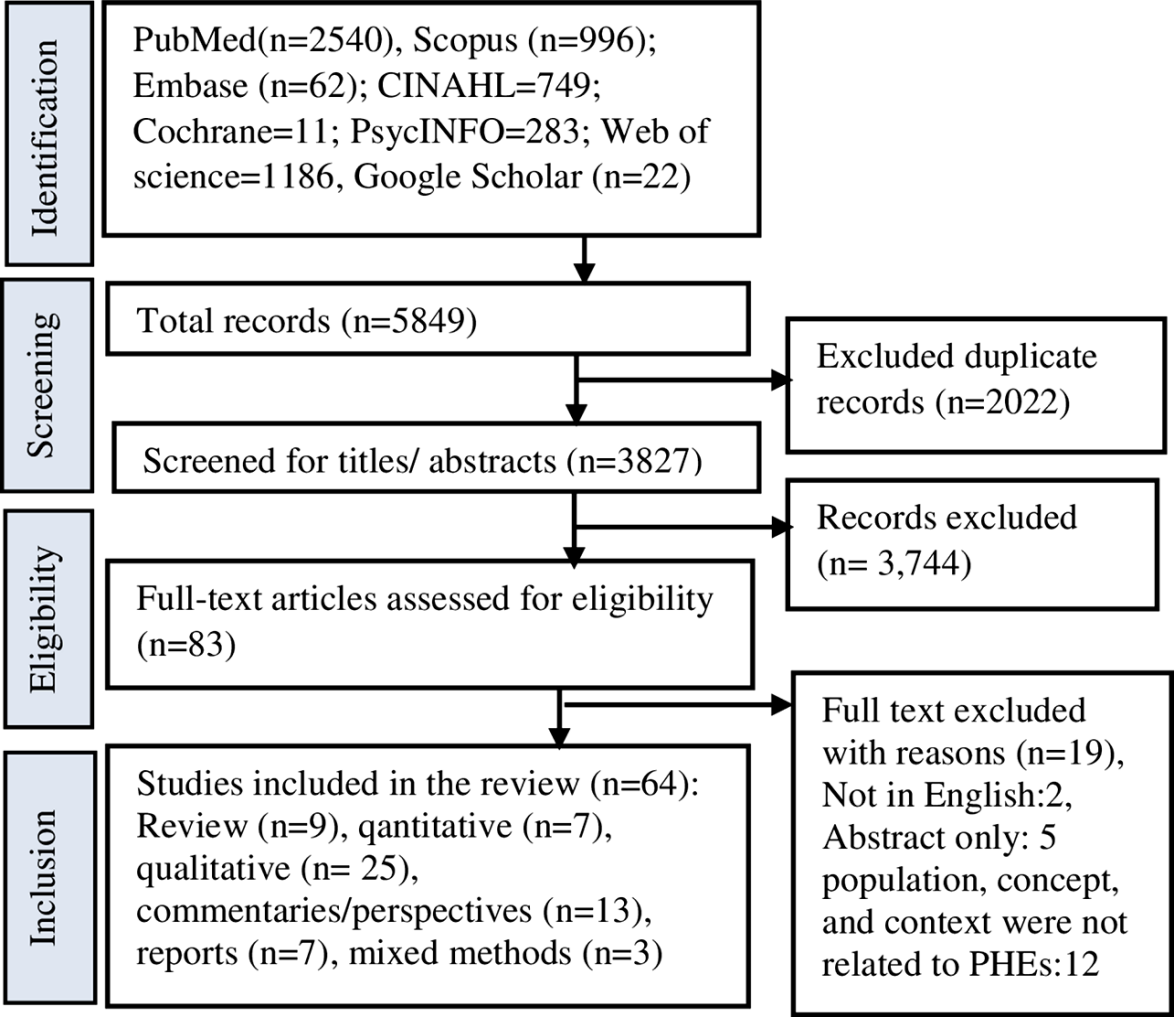
PRISMA-ScR flow chart showing the selection of studies for the review

Figure 1 presents the selection studies for the review (Fig. 1). The search strategy returned 5849 articles/studies, including the grey literature and forward citation searches. After duplicates were removed, 3827 articles were screened for relevance based on title and abstract, where 2022 articles were excluded, leaving 83 articles for full-text screening. A further 19 articles were excluded after the full-text screen. A total 64 studies were included in the final review. Of 64 studies, 47 were related to outbreaks, mostly explaining the COVID-19 pandemic. Seven studies explained complex emergencies (e.g., concurrent conflicts, outbreaks, and disasters), six discussed conflicts, and four explained disasters caused by natural hazards.

### Main themes from the included studies

Several themes were identified regarding the impacts of PHEs, and lessons learned while responding to those PHEs. Table 2 presents themes on preparedness, impacts, and response of PHEs towards health security.

**Table 2:** Preparedness, impacts and lessons learned in responding to public health emergencies

PHE stage/Themes	Drivers (enablers and barriers) of public health emergencies	Countries / Regions
Preparedness		
Preparation and surveillance systems	Decentralised health system governance [25], intercity coordination [26], surveillance and daily summary reporting [27], preparedness, planning, surveillance, and proactive response with PHC services [28–30]. Shortage of staff and supplies, poor preparation for emergencies, missing standard operating procedure [31–36], poor transportation and communication [33–36], inadequate preparedness [37], knowledge gaps on contextual governance [13], poor understanding of PHC by non-health sector [16, 38], lack of planning [39].	Cameroon, Congo, Mali, Nigeria, Iraq, Ecuador, Indonesia, India, China, Japan, Central African Republic (CAR), Isreal
**Impacts**		
Increased health needs	Lack of shock absorption and adaption capacity to respond to shocks [31, 32], lack of health services [33, 40], increased epidemic [41], collateral effects [42], increased displaced. Host populations [43], weak capacity distribution of supplies, unavailability of quality services [42, 44], and postponed routine care in PHC [33, 45].	Cameroon, Congo, Mali, Nigeria, Iraq, Eastern Medicterrain Region (EMR), Libya, Yemen
Constraints in service delivery	Lack of trained workforces [46], failure of coordinated support overburdened hospitals [47], lack of primary care [45], high priority of hospitals services [48], health workforce workload [49, 50], an increase of infections [15], the hotspot of epidemics but blind spot of PHC services [28, 37], the spread of Ebola [43], increase of pre-existing diseases [34], reduced availability of services [45, 51].	Italy, Australia, Brasil, Malawi, SSA, CAR, Ecuador
Multiple impacts on building blocks of health systems	Inadequacies of service provision [47, 51, 52], isolation, lockdown, restriction [53, 54], collateral damage, interruption of service [53, 55–58], post-disaster disease outbreaks [33, 34], shortage of workforce and heavy workload [33, 34, 47, 50, 51, 55–58], reliance on short term staff [46], poor data quality [56], poor resource coordination and readiness [29, 52, 59], unavailability of digital tools [16, 27], poor digital interoperability remote areas [60, 61], poor partnerships, inadequate investment [56], market-oriented health systems [62], failure of global health response [63, 64], corruption in procurement, chronic under-investment [44, 60], and poor investment from private sector [43].	Malawi, sub-Saharah Africa (SSA), South Africa, Germany, Ecuador, Australia, Japan, LMICs, Yemen
Increased health inequities	Exacerbated the existing disparities [39, 62, 64], increased geographic inequities [31], and poor global response to reduce inequities [12, 62].	Cameroon, Congo, Mali, Nigeria.
**Responses**		
Integrated public health and primary care	Investment and reorganisation of PHC systems [28, 48, 51], coordinated public health and primary care [38, 65, 66], strengthened health systems [45, 67], and ability to adapt to the situation [58]. Cumulative service capacity [37, 68]. Implementation of PHC for equity [13], mobilisation of frontline workers [38, 59], technological innovations [55], caring for vulnerable populations, and use of information technology [55, 69]. Empowering PHC institutions for primary care [29], developing facility-specific preparedness plans standard operating procedures [36].	CAR, Brazil, Malawi, Germany, Cameroon, SSA, Liberia, India
Multisectoral actions	Linkages between policymakers, community based organisations, NGOs, and private sector [13], community resilience, satisfaction, and confidence [16, 30], Engaging stakeholders in planning [52, 64], multisectoral actions for prevention of pandemic [54], community collaborations [30, 70], multi-sectoral and comprehensive provincial pandemic and economy [56], “One Health” approach [29, 63], multisectoral actions for non-health sector response [71], multisectoral coordination, integration of fragmented approaches [72].	Israel, Thailand, South Africa, Cuba
Communication and partnership	Strategic partnerships of international organisations [43, 49, 73, 74], rebuilding coordination and communication networks, [27, 47, 67], humanitarian response, maintaining and outreach or mobile clinics [31, 42, 68], hospitality towards displaced populations [40], regional forums, institutions [26, 72], global initiatives and cross-country lessons [71, 75].	Sierra Leone, China, Italy, Japan, Yemen, Cameroon, Congo, Mali, Nigeria, EMR
Use of digital tools	systems shift to using new technologies and digital tools [26, 45, 49, 50, 76], digital consultation communication in remote consultations [53, 55, 68, 76], learning tools [60], use in data collection and supervision [44], reached vulnerable groups, optimisation of workers, and telecare [50, 77–79], set alarm systems, generate real-time, monitoring and evaluation [50, 77, 78], sharing information and communication [39, 80], technology informed multidisciplinary care [67], DigiTech in data processing, research [43, 71].	China, Australia, LMICs, Dubai, SSA
Multidisciplinary health providers	service delivery by CHWs [44, 55], family health teams in flu assessment [67], VHVs’ role in identification and monitoring of returnees [81], CHVs’ role in child health services, control of Ebola and malnutrition [42], integrating health-care system to enhance the public workforce practitioners [26], redefining training plans for safe working environments [51, 56, 82], intrinsic motivation and self-initiative [58].	Yemen, SSA, Thailand, China, Malawi, South Africa, Germany
Planning for resilient health systems	organization and pre-emptive planning [52, 57, 58], context-specific priorities, resilient health infrastructures [83], institutionalizing health programs [69]. Provision care with resilience in difficult conditions [42, 52, 57, 58], decentralized Brazilian health system [65], integrated health-care system [26], national intersectoral government plan [54], adaption and restricting or transformative activities [32], national consensus on contingency plan [82], monitoring to avoid unnecessary contact [66, 84], reorient health interventions [28], incorporating preparedness exercises [43].	Germany, Yemen, Brazil, China, Iraq, European Union (EU), CAR

### Preparedness

Preplanning, ensuring monitoring and surveillance of PHEs are key to reducing the potential consequences of PHEs. There were several examples of preparedness and surveillance in different contexts.

#### Preparedness and surveillance

Health systems have faced several challenges in disaster preparedness. Those challenges included a shortage of staff and supplies, poor preparation facilities for emergencies, lack of electricity backup, and missing standard operating procedures and policies [31–36]. Other hindering factors for disaster preparedness were poor transportation, inadequate communication, and incident command systems [33–36]. For example, Cameroon’s weak PHC systems with inadequate preparedness for PHEs hindered the health response systems and recovery strategies during and following the COVID-19 pandemic [37].

In conflicts and disasters, critical knowledge gaps and context-specific challenges in health systems (e.g., governance, financing, workforce, accountability, and service coordination mechanisms) affected the PHC implementation [13]. In natural disasters, poor understanding of PHC of stakeholders from non-health sector and the health sector’s silo approach also influenced integrated disease management [16, 38]. Lack of planning and defining the roles of professionals and disarticulating actions with real needs hampered the PHC services delivery to the COVID-19 pandemic-affected populations in many countries [39].

Nevertheless, there were some successful examples of preparedness for PHE responses. For instance, Indonesia’s decentralised health system governance and strengthening (e.g., national action plans for health security, preparedness planning and exercises) enhanced emergency preparedness strategies [25]. These strategies included mandatory minimum standards at a local level, integrated with a national disaster management system, decentralised contingency plans, and simulation exercises for potential future PHEs. In China, the experience of the city of Shenzhen in coordinating their health care systems’ preparedness helped other cities to enhance and deal with response capacities in future emergencies [26]. In Japan, daily reporting of post-disaster disease surveillance was critical for tailoring responses to local settings, establishing support networks, and integrating resources [27]. In addition, the proactive reorganisation of PHC services paved the way towards increased pandemic preparedness, planning, surveillance and responses for future health system shocks [28–30].

### Impacts of public health emergencies

The PHEs have direct impacts (e.g., interruption of supply chain and health service delivery) and indirect (e.g., collateral impacts including damage to infrastructure, road networks, and communication systems). These impacts led to creating structural and health inequities.

#### Increased health needs

During the armed conflict, there have been increasing numbers of internally displaced persons and refugees, leading to overcrowding and overburden for existing systems and service delivery. For example, in the Democratic Republic of the Congo (DRC), conflicts further triggered an increase in Ebola cases that overburdened health systems and increased health service needs [33].

Displaced populations due to armed conflicts need health services that could lead to overburdened health systems, interruption of health service delivery, and challenges in the implementation of PHC. Some implementation challenges of PHC in PHE contexts including armed conflicts covered under-preparedness and lack of shock absorption capacity in public sector, limited ability to provide services, poor adaptation to shocks, lack of restructuring of damaged facilities, limited resilience to conflict difficulties, and rebuilding community trust in the public sector [31, 32].

Furthermore, displaced and host populations in conflict-affected settings both lacked public health services and experienced further exposure to the risk of infections and mental health issues [33, 40]. Those affected populations had poor access to hygiene and sanitation (e.g., access to safe water) and lacked access to PHC services [33, 40]. For instance, in Libya, the impact of conflicts was structural damage to health facilities, shortage of medical supplies, lack of security of PHC staff, and lack of communication, all of which collectively led to an increase in neglected and orphaned children and the emergence of unusual infections [41]. In DRC, there were no integrated community mental health services despite increased mental health problems due to armed conflicts [33].

Armed conflicts had collateral damage in the context of fragile health systems that further influenced the access and delivery of health services. For instance, in Yemen, the ongoing war has increased cholera outbreaks affecting the health system to meet those health needs [42]. In armed conflict-affected regions such as the Ebola epidemic in Guinea, Sierra Leone, and Liberia, health systems became fragile, which deteriorated the provision of essential public services to both displaced and host populations [43]. Furthermore, conflicts also affect care-accessibility by interrupting the supply chain management and short-term programs [42, 44]. Factors affecting health care delivery in conflict affected settings included lack of integrated community health, difficulties in travel, poor supervision and monitoring, threats to health workforces, weak supply chain management capacity, unavailability of quality services, politicization of aid, and increased costs of care [42, 44]. In addition, civil instability and natural disasters resulted in individuals abandoning or postponing routine care, including mental health services [33, 45].

#### Constraints of service delivery

Several health system factors create difficulties in health service delivery in PHEs. For example, in Australia, a lack of trained PHC workforces increased the risk of transmission of COVID-19 in remote areas [46]. Failure of coordinated support in PHC services overburdened hospital services and overcrowding increased the chance of nosocomial infections in Lombardy, Italy [47]. Furthermore, disturbance in PHC systems increased cases without PHC services in preventing and controlling outbreaks in Brazil [48]. For example, in Malawi, key health services were interrupted, reducing clients attending facilities in PHEs [51]. Instead, the priority was given to the hospital sector, resulting in the poor and ill-equipped first point of care to protect staff and patients from infection and provide primary care [45, 48].

Furthermore, PHEs resulting from catastrophic events impacted the roles of the health workforce (e.g., task-shifting responsibilities and changes in the scope of work, financial strains, daily uncertainties, and stress). They hindered the delivery of primary care services [49, 50]. Neglected or postponed essential care, lack of gatekeeping, limited capacity, and weak integration between medical care and public health influenced factors of delivery of patient care services [49, 50]. In Sub-Saharan Africa, insufficient investment in health systems and increased pandemic is a reminder that non-communicable diseases, which are increasingly prevalent, are closely interlinked to the burden of communicable diseases that exacerbated poor health outcomes such as morbidity and mortality [15].

Countries like Cameroon and the CAR had hot spots of emergency outbreaks but lacked PHC services as blind spots of outbreak response [28, 37]. New epidemic outbreaks in Ecuador were exacerbated by a lack of preparation, poor information on health indicators, a shortage of resources (personnel and physical infrastructure), poor PHC services, and a sharp increase in pre-existing diseases [34]. During the pandemic, health systems had the availability of comprehensive services and adaptation to unique demands of resources. In contrast, people’s lives and the economy were impacted by service users’ discrepancies between reported behaviour and practice (e.g., consistent use of masks) [45, 51]. Furthermore, political disputes and constraints of financial resources in strengthening the PHC system hampered and obscured primary care, which influenced the health systems’ capacity to address health needs and effectively implement infection control protocols [28, 37]. In the case of Ebola response and infection control in Guinea, Sierra Leone, and Liberia, conflicts weakened primary care systems and contributed to the fast and rapid spread of diseases [43].

#### Multiple impacts on building blocks

PHEs broadly – and COVID-19 specifically – impacted all building blocks of health systems. Firstly, health systems lacked facility readiness for health services, including lack of material resources (e.g., soap, hand sanitiser, water, masks, equipment, test materials, and staff), inadequate infrastructures (e.g., lack of equipment and space), difficulties with procurement of test kits and turn-around times, neglected PHC systems, poor health service provisions, and inadequate management of cases and physical distancing [47, 51, 52].

Second, COVID-19 hindered the delivery of PHC services and health care deficiencies due to continued isolation, lockdown, and restriction of critical services, especially in remote areas in Australia [53, 54]. The COVID-19 pandemic amplified the fragility of existing systems, caused a de facto lockdown and associated collateral damage, and disrupted traditional delivery models in Sub-Saharan Africa and South Africa [53, 55–58]. Following natural disasters, damage to health infrastructure has contributed to the eruption of post-disaster disease outbreaks in Ecuador and Ebola-affected countries in Africa [33, 34].

Third, there was an impact on the health workforce, including a shortage of clinical workforce; fatigue and stress from heavy workloads, stigma, worries of infection, burnout, grief; and lack of training of junior doctors [33, 34, 47, 50, 51, 55–58]. For example, during the COVID-19 pandemic, the Australian health system experienced an acute shortage of health workforce (e.g., nurses) and relied heavily on short-term workforce such as fly-in, fly‐out/drive‐in, drive‐out staff to provide care in the country’s remote regions [46].

Fourth, during the COVID-19 pandemic, health systems response failed to consider or deal with their fears and ability to care for patients when confronted with poor data quality and inappropriate administrative decisions on self-standing field hospitals and information gaps [52, 56]. Modern health care systems are highly vulnerable to the unavailability of digital communication tools [16, 27]. Implementing remote consulting was challenging due to poor digital interoperability (e.g., lack of digital infrastructure and resources). High data or airtime costs affected upscaling, training, and providing care and health education [60, 61].

Finally, current global health systems are guided by the market-oriented political economy of health systems, which created difficulties in providing PHC services in a pandemic [62]. Community engagement and buy-in are critical for maintaining service provision in emergency contexts. For example, South Africa faced challenges in COVID-19 response related to poor partnerships between health systems and communities, as well as inadequate investment in PHC from the private health sector [56]. Lessons learned from past and current pandemics show that the failed responses of global health systems might create difficulties in handling future pandemics [63, 64]. Health systems also struggle with poor governance, including increased corruption in procurement at the country level [60]. Drivers of poor governance included chronic under-investment, insufficient workforces, lack of coordination in plan and funding programs, inflexible billing and record-keeping systems, and limited community awareness [44, 60]. As a result, the private sector may not invest in future PHEs responses and be disincentivized from investing in such opportunity costs in shifting resources away from commercial projects [43]. Poor capacity, including lack of resources, infrastructure, and reactive responses, for PHEs threatened the realisation of universal health coverage. Factors influencing poor public health response of PHCs were lack of coordinated efforts (primary care and public health), lack of resource coordination, and poor readiness of public health institutions [29, 52, 59].

#### Increased health inequities

Impacts of PHEs and globalization in trade and commerce also influence structural determinants of health. Unequal distribution of social determinants of health contributes to new inequities and increases existing equity gaps among priority populations. PHEs reduce access to services, especially marginalised people, and disproportionately exacerbate structural (e.g., education and wealth) and geographical disparities that lead to increased health inequities [31, 39, 62, 64]. Other impacts of PHEs (e.g., outbreaks) included the digital divide (e.g., exclusion of some populations due to digital and Wi-Fi access), unequal use of services offered, and compounded, long-standing health discrepancies [39, 62]. The unpreparedness of professionals using digital technologies and fragile articulation between remote and face-to-face modalities increased health inequities throughout the COVID-19 pandemic [39]. Global responses failed to reach the purpose of policymaking for pandemic responses, while neoliberal governance approaches created increased inequities that further challenged achieving UHC [12, 62].

### Response to impacts of PHEs

Lessons learned to respond to PHEs were preparation, integration of primary care and public health, multisectoral action, use of digital systems, communication and partnership, and building resilient health systems.

#### Integrated public health and primary care

Effective PHE response requires integrating public health functions and primary care. Implementing the PHC approach linked with social determinants of health was effective, and strategies included investment in public health systems, reorganisation of PHC services, and training front-line providers [28, 48, 51]. Furthermore, coordinated public health and primary care efforts could implement the vision of PHC and values for health development [38].

The provision of primary care can ensure the prevention, protection, promotion, and treatment of illness in individuals and communities that improve the social and economic indicators [65, 66]. Lessons learnt from the pandemic response were strengthening health systems for primary care in complex situations by connecting public health and primary care and coordinating resources for services—a strong ability to adapt to system resilience [45, 58, 67]. In the pandemic response, health systems’ involvement in PHC actions ensured a continuum of service with the cumulative capacity to meet emerging health needs in the communities [37, 68].

In a pandemic, implementation and investment in public health and primary care improved equity and access, harmonisation, and synergise in building healthy societies responded to emergencies through mobilisation of frontline service delivery healthcare performance, accountability of health systems and health outcomes [13, 38, 59]. For example, in Sub-Saharan Africa, the COVID-19 pandemic exposed an opportunity to implement a community-orientated primary care approach and apply the long-term benefits of technological innovations [55]. Strategies adopted for primary care and public health included integration of community-based activities, screening and testing, reorganisation of health services, maintenance of essential and emergency health services, caring for vulnerable populations, use of information technology, reframing training opportunities, and empowering PHC institutions in primary care [29, 55, 69]. In floods, preparedness and response strategies were developed for facility-specific preparedness plans with standard operating procedures and identified a chain of command [36].

#### Multisectoral actions for impact responses

Strengthening linkages among stakeholders – policymakers, civil society, non-governmental organizations, community-based organizations, and private sector entities – enabled equity-informed financing models and health systems governance frameworks that differentiated from more discrete service-focussed primary care [13]. Community responses included community engagement, collaboration, and networking to address the collateral impacts of emergency events. Suburban communities reported community resilience, satisfaction, and confidence and repositioning approaches in healthcare services to meet people’s needs in the COVID-19 pandemic in Israel [16, 30]. Engaging public health stakeholders in community planning improved primary care practices and built trust between institutions, communities, and health systems [52, 64]. In Cuba, multisectoral actions were incorporated into prevention and control that helped mitigate the COVID-19 pandemic’s impact [54].

Mechanisms of empowering agencies to encounter the invasion of a global pandemic were community collaborations, social networks, social capital, and the role of PHC in minority communities in emergency and routine care [30, 70]. In South Africa, comprehensive multi-sectoral actions effectively addressed health system fragilities and saved lives and the economy during the COVID-19 pandemic at the provincial level [56]. Additionally, strengthening and implementing the “One Health” approach and empowering PHC institutions enabled countries to meet pressing needs in pandemic preparedness [29, 63]. Such approaches are in-line with the Sustainable Development Goals, which highlight how essential development actions of human life and multi-sectoral cooperation can improve multisectoral coordination, integration of fragmented approaches, ensure knowledge exchange and implementation, and respond to the fragility of the health system for improved populations’ health and well-being [71, 72].

#### Communication and partnership

Coordination and communication, including communication and coordination among stakeholders and sectors and strategic partnerships, enhance health service delivery in PHEs. The role of partnership among international organisations became a tool for procurement, deployment, supply chain management, mitigating stockouts, ensuring cost efficiencies, provision of medical supplies and healthcare infrastructures development in the Ebola outbreak in Sierra Leone, Guinea and Liberia [43, 73, 74]. Strengthening human and technical resources, rebuilding networks and alerting evacuation centres avoided overcrowded hospitals by protecting patients and providers in PHEs such as the COVID-19 pandemic and natural disasters (e.g., earthquakes) [27, 47, 67].

Funding and technical responses from humanitarian agencies reduced the opportunity costs and decreased the severity of the crisis [43, 74]. In fragile and conflict-affected settings, health care assessment, including situation mapping of local characteristics of disease transmission, demography, public health services organization, and health system’s capacity and financing and actions were used to provide health services in PHEs, maintain and function facilities, and deploy outreach or mobile clinics and teams [31, 42, 68]. Displaced populations from the Yemen conflicts are relocated to some Eastern Mediterranean Region such as Lebanon, Iran, Pakistan; however these displaced populations across the region living outside camp settings are exposed to increased public health risks, including infectious diseases due to overcrowded living conditions, and varying degrees of access to PHC services [40]. The role of partner organisations and global initiatives such as regional forums, institutions, and policy technocrats played a crucial role in cross-country sharing of lessons learned and in procurement of resources to improve efficiency and regional sharing in policy development and implementation. Partnerships and collaboration allowed for ensuring essential health services, reaching unserved populations, protecting against financial risk, increasing satisfaction, and improving health security and coverage of health services [26, 71, 72, 75]. Multi-country mechanisms, multilateral technical cooperation, and regional forums facilitated and maintained essential health services by leveraging resources for pooled procurement and helped prepare for future health crises [73, 75]. International declarations on digital health also called for employing advanced technologies for health in data processing, research, and development and clarifying approaches for regulatory pathways for new tools [43, 71].

#### Use of digital tools

The use of digital tools and systems was found to be effective in responding to the impacts of PHEs. For instance, the recent responses to PHEs shifted towards using new technologies [49, 50]. Service delivery approaches using digital tools (e.g., e-health, e-mail, and virtual consultation) increased completion rates (e.g., older working-age persons) [26, 45, 76]. Similarly, in Dubai, telemedicine service increased by 86% in general and COVID-19 consultations [50].

During the COVID-19 lockdowns, a surge of digital consultation expedited service delivery, improving access to primary care, and facilitating the provision of services in remote areas [53, 68, 76]. In addition, digital technology supports health staff in receiving and applying skills, and helping service users (e.g., rural areas, rigid work schedules, transportation problems, complex health problems) in behaviour change activities [60, 76].

In Yemen, mobile technology was used in supervision, data collection, pre-positioning buffer stocks in the community, and communicating risk-reduction measures such as avoiding travel during peak violence/crisis, safety training, and risk communication [44]. Furthermore, digital tools were adopted to develop early warning systems for disasters, generate real-time information, and monitor and evaluate [50, 77, 78]. Implications of digital systems bolstered PHC services to reach vulnerable populations, enabled clinicians to provide and maintain necessary public health measures, optimised providers’ work, and created user-centred designs with sustainable and scalable programs to meet the needs of affected populations [50, 77–79].

Digital tools can be used in learning and communication in remote and in-person work or conducting remote consultations [53, 55]. Evidence suggests that system efficiency was improved in disasters by addressing economic, social, and geographical constraints [39, 80]. Digital strategies were adopted for information and communication to optimise the organization of quality of care, strengthening of continuity of care, cultural accessibility, and appointment time [39, 80]. Coordinated multidisciplinary primary care teams employed digital solutions to deliver essential services in PHEs [67].

#### Multidisciplinary health providers

In conflict-affected areas, service delivery by community health workers played a vital role in using community resources and delivering medications to people with chronic conditions [44, 55]. Care providers took public health responsibilities, worked closely, and played a ‘sentinel’ surveillance role in identifying re-emerging COVID-19 cases in China [49]. Integrated, interdisciplinary family health teams provided flu assessment centres and provided public health information about infection control and antiviral medication. Furthermore, these teams provided timely, coordinated, and comprehensive responses to public health emergencies, offering a promising new direction for healthcare organisations [67]. In Thailand, the timely mobilisation of trusted village health volunteers identified and monitored returnees and was used in the surveillance of the COVID-19 pandemic, including the referral of symptomatic patients to hospitals for care [81]. This model helped to contain the pandemic without countrywide lockdown and mass testing [81]. Mobile clinics with networks of community health volunteers (personal with limited training, work voluntarily, and connect community and formal health systems) in conflicts in Yemen met urgent needs, including specific child health services, control of the cholera epidemic and treatment of acute malnutrition with precedence of other services in the epidemic [42]. Community containment of the COVID-19 epidemic in Shenzhen, China, was possible by integrating the health care system to enhance the public workforce in PHC [26]. The COVID-19 pandemic highlighted the need to redefine the training plans for safe working environments and training (e.g., psychosocial support to manage impacts) [51, 56, 82].

#### Planning for resilient health systems

Adequate organization and pre-emptive planning mitigate barriers to quality patient care, support disease surveillance and contact tracing, and optimize limited resources (e.g., personal protective equipment for new public health emergencies, testing, and the role of workforce responding to threats) [52, 57, 58]. Strengthening ing of the health services in conflict-affected settings and delivering equitable PHC services require context-specific priorities, engagement of community, non-health sector collaboration, and developing resilient health infrastructures under social crises response to health system building blocks [83]. For example, in Liberia, the health system was designed to better prepare for future shocks through institutionalizing standardized community health programs with fit-for-purpose and incentivized community health assistants [69]. However, the experience of PHC providers and understanding of workers effectively understood the gaps in planning and management displayed in health care provision care with notable resilience working in difficult conditions in PHEs [42, 52, 57, 58].

Decentralization has the potential to establish re-organization and strengthen health system for PHC and orientation of health interventions [28, 65]. For example, in China, an integrated health care system employed core strategies for improved emergency responses and delivery of health services [26]. In Cuba, a national intersectoral government plan was adopted in the pandemic response, including research in diagnosis and case tracing use of universal protocol for prevention and treatment [54]. Planning response of health institutions was effective in responding to future emergencies [57, 58]. In Iraq, conflict-affected governorates implemented resiliency strategies such as absorption, adaption, restriction, or transformation activities [32]. In Nepal, National Coordination Centers for PHEs were established for rapid and efficient responses that developed a national consensus on contingency plans, use of data and capability resources, bioethical response and respect for people’s values, and truthful communication systems [82].

Developing monitoring and warning systems can detect hotspots of PHEs while identifying blind spots of PHCs services in affected areas. Monitoring and prescriptions also avoid unnecessary contact by improving early warning and detection systems, involving trained workforces, and incorporating preparedness exercises [43, 66, 84].

## Discussion

This study synthesized several themes on preparedness, impacts and response to PHEs. Major PHEs identified in the review were related to armed conflicts, disasters, and outbreaks. The impacts of PHEs in health systems were increased health needs, constraints in access to and delivery of health services, impacts in health system building blocks, and increased health inequities. Most of the studies were from LMICs that went through several PHEs while health systems had poor preparedness and response strategies. The themes of PHEs response were the delivery of integrated public health and primary services, communication and partnership, digital tools, multisectoral actions, utilization of multidisciplinary health providers, and developing resilient health systems.

### Immediate health sector response

Addressing the immediate health impacts of PHEs is to ensure health services are accessible to affected populations. Firstly, it is important to identify the hotspots of affected areas and populations. Second, the health system must ensure PHC services and primary care at the point of care [45]. Third, healthcare assessment in PHEs requires the identification of local characteristics of affected populations (e.g., disease transmission, demographic distributions) and health system readiness (e.g., public health services organization and planning) [31, 68]. In addition, understating short- and long-term impacts in delivering essential services, quality assurance, and provision of health workers is also vital to the immediate response impacts of PHEs [64].

In the context of PHEs in LMICs, the role of CHWs is pivotal to providing immediate response and implementing PHC services at local health facilities. Community health workers require decent working conditions, training, and continuing education to build their capacity. The effective utilisation of community health workers is centred on the premise that PHC can work in culturally competent and community-oriented ways [61, 84]. Furthermore, multidisciplinary primary care teams can identify vulnerable populations needing medical and social outreach services in emergencies [84]. Additionally, public health institutions can play an indispensable role in mobilisation of community health workers in providing services in PHEs [29, 72].

Partnership and coordination are also vital to ensure PHC services in the PHE context. Integrating PHC and public health in PHEs and implementing with partnership (local teams and organisations) can help to cope with current and future waves of pandemics [45, 67]. The social networks and engagement of private sector and local resources mobilisation (e.g., local stakeholders) help to understand larger societies and allow the piloting of novel solutions [53, 70]. In addition, the role of non-governmental organizations in current and future epidemics could support the development of policy tools in partnership in response to PHEs [74]. Furthermore, post-PHEs, mental health problems are more likely to emerge in affected populations, which warrants the integration of mental health into existing health systems [33].

The implementation of digital service approaches (e.g., telehealth, video consultation) has been increased in PHE contexts and offers an effective approach for improving access to health care. Adaptable digital tools have enabled the implementation of PHC and provided solutions in health emergencies [50, 53, 78]. The positive impacts and advantages of technologies could be vital remote strategies for primary care quality to ensure knowledge exchange and implementation of PHC [39, 72]. Innovative tools and technologies for digital health are transforming the culture and practice of public health and improving access to PHC services in remote areas [16, 53]. Digital health solutions in PHEs can consider interoperability of existing systems, provision of medical supplies, training to staff, managing demand (e.g., risk communications, prioritisation of pandemic response efforts) linked to vulnerable populations) [74, 84].

The COVID-19 pandemic warrants a sense of purpose to health policymaking and demonstrates differences in the organization of fast and urgent implementation of digital strategies in PHC worldwide [39]. Adaptive and transformative measures can be taken at PHC practices, setting up outpatient infection centers, and coordination processes (i.e., actively transferring knowledge, integration in crisis management teams, and regional strategic efforts). Responding PHEs are required to integrate into public services by developing response capacity (through information and communication technologies) and managing challenges through evidence-based planning [12, 39].

### Impact minimisation through multisectoral actions

The PHEs can have spillover effects requiring multisectoral actions. Developing resilient and integrative healthcare systems requires analysing structural determinants and multisectoral actions [47, 72]. Building resilience for future shocks and strengthening PHC can be viewed beyond the health systems lens [69]. Coordination, leveraging partnership support, using a systematic approach to inform policy shifts and strengthening community engagement potentially trigger multisectoral actions in PHE contexts [69].

Effective policy response to absorb and adapt to the impacts of health emergencies to promote health and wellbeing [15, 16]. Addressing the complexities of conflict conditions underscores the importance of PHC development in promoting health [83].

Spill over or collateral impacts of PHEs interrupt the functioning of the public health ecosystem, including sanitation, transportation, communication, and supply chain systems and services. Responding to those collateral impacts is vital to minimise the short-term and long terms effects of PHEs [85]. In addition, PHEs response through multisectoral actions could go beyond affected populations and risk populations with wide geographic and population coverage, a blend of public health and primary care, and referral services for higher care [86].

Short- and medium-term multisectoral strategies could facilitate local resource mobilisation, addressing immediate impacts and reducing the potential of the emergence of post-PHEs disease outbreaks. Such strategies include establishing coordination mechanisms such as institutional arrangements such as activating working groups; non-medical responses for ensuring call centres for the response, supply of foods and accommodation logistics; and establishing primary care facilities in outreaching settings [87]. Other approaches include the implementation of PHC, coordination and communication of sectors, use of local strategies, resource mobilisation, and implementation of population health interventions [88].

Long terms strategies in PHEs aim to reduce long-term impacts by reducing the emergence of NCDs, malnutrition, or post-traumatic disorders. These actions can be implemented by involving multiple stakeholders and non-health sectors in the PHEs context. The urgent health needs are further underscored and compounded by pressing economic, demographic, and climate issues [16]. Perspectives from other stakeholders in the PHC system are also fundamental in multisectoral planning and developing resilience in primary care [58]. Government and organizational support are required to facilitate the program’s expansion through digital systems [60].

### Preparedness and surveillance for future PHEs

The health system requires preparing a national health system (e.g., monitoring, surveillance, mitigation and response). Therefore, maintaining and building PHC systems and strengthening preparedness, including health workforce preparedness (disaster preparedness training) is vital for responding to current PHEs and preparing for future events [35, 84]. For this, national needs and actions require prioritising the aspirations of PHC in PHEs context [42]. In addition, the PHEs (e.g., pandemic) provided an opportunity for international communities of public health professionals and institutions to re-imagine health systems approaches beyond classical models and reconquer constraints for a healthier future [12].

Solely using long-term surveillance to map crisis hotspots is a blind spot in delivery of PHC services [28]. Response preparedness for current and future PHEs requires implementation science, investments, and strategies to bridge the persistent evidence-practice gaps characteristic of long-term surveillance systems [64]. In addition, addressing outbreaks (e.g., COVID pandemic) requires government will and cooperation with adequate social services [62]. Understanding human-environmental impacts are essential during the pandemic, which offers insight into the emergence of future pandemics and the climate crisis [89]. The pandemic created opportunities to innovate ways to build a resilient data collection system with a warning and response system to recruit local clinicians and train personnel for diagnostic tests, drugs, and vaccines [43, 80].With surveillance actions, the community followed up on recovered patients, lab tests, care, and treatment [53, 54].

In the digital era, global health is evolving, aiming to explore needs, and offering equitable health services [71]. The pandemic created momentum, pivoting health towards PHC and equity outcomes; thus, a revolution in health system governance is required to re-examine the architecture governance, funding, and sustainable response to PHEs [15, 16]. For this, global consensus should focus on therapeutic resilience, the use of health care resources, and sustainable and effective delivery of PHC services [66]. The global health agendas (e.g., global health security and universal health coverage) warrant a synergistic solution by leveraging resources (multi-country pooled procurement, enabling countries to prepare for quality health services, and affordable essential medicines) [63, 73]. Global institutions should have enough authority and funding to coordinate decision-making (for global warming and response systems) [43]. Meanwhile, national and global perspectives are integral to engaging public health approaches to reduce the impact of climate change [89].

### Policy and research implications

Most of the studies included in this review focused on the acute impacts of PHEs and their responses. Responding to and mitigating these acute impacts is important. Still, silent PHEs, such as the impact of economic recession, mostly in high-income countries, as well as famine and malnutrition in LMICs, are also important global health security threats. Therefore, research needs to focus on the chronic impacts of global health security issues. Acute and chronic PHEs lead to increased vulnerabilities and equity gaps. Responding to chronic PHEs requires macro-level and long-term strategies, utilization of the architecture of global institutions and governance systems, and global monitoring and surveillance mechanisms.

### Strengths and limitations of the study

This review presented integrated findings from studies using a range of designs and methodologies and explained findings relating to PHE preparedness to responses. Limitations of this study include no quality appraisal of the individual study in the review and no inclusion of studies published in languages other than English. However, the purpose of our review was to synthesize the available evidence rather than grade the evidence. We utilized a systematic scoping review methodology to review available evidence following PRISMA-ScR guideline [18, 19] and taking references from the previous scoping review [21, 22]. Additionally, we searched eight databases and comprehensively searched studies important studies are included to include the most relevant. We found studies related to three PHEs (e.g., conflict, outbreaks and natural disasters); however, other catastrophic events such as financial hardships, economic recession, and famine are also important events that can affect public health in the form of indirect impacts on public health services. Therefore, further research should consider other emergencies with public health implications.

## Conclusions

Public health emergencies can have multiple health and collateral impacts in countries with fragile health systems, poor preparedness, and inadequate surveillance mechanisms. Health systems efforts need to focus on preparedness to absorb the shocks from PHEs, respond to them and adapt to future emergencies. Some potential strategies to respond to impacts could be ensuring health services to address health needs unique to emergency contexts, monitoring and surveilling of outbreaks post PHEs, and operationalizing multisectoral actions to solve the collateral damages. In addition, risk assessment, disaster preparedness, and setting alarms using digital systems could mitigate future health emergencies. Responses of PHEs require the adoption of three-pronged strategies: preparedness (e.g., surveillance, health system readiness); response to immediate health impacts (e.g., improve acute access to health services); and mitigation of collateral or spillover effects through multisectoral policy and actions.

### Electronic supplementary material

Below is the link to the electronic supplementary material.

Supplementary Material 1

## Data Availability

All data generated or analyzed during this study are included in this published article [and its supplementary information files].
