# Supplementary material for: Preparedness, impacts, and responses of public health emergencies towards health security: qualitative synthesis of evidence
Source: Arch Public Health. 2023 Nov 30;81:208. doi: 10.1186/s13690-023-01223-y (PMC10687930; doi:10.1186/s13690-023-01223-y)
Supplement: Supplementary file 1 — Supplementary Material 1 [file 13690_2023_1223_MOESM1_ESM.docx]

**Supplementary information, Table S1: Preferred Reporting Items for Systematic reviews and Meta-Analyses extension for Scoping Reviews (PRISMA-ScR) Checklist**

| SECTION | ITEM | PRISMA-ScR CHECKLIST ITEM | PAGE # |
| --- | --- | --- | --- |
| TITLE | | | |
| Title | 1 | Identify the report as a scoping review. | 1 |
| ABSTRACT | | | |
| Structured summary | 2 | Provide a structured summary that includes (as applicable): background, objectives, eligibility criteria, sources of evidence, charting methods, results, and conclusions that relate to the review questions and objectives. | 1 |
| INTRODUCTION | | | |
| Rationale | 3 | Describe the rationale for the review in the context of what is already known. Explain why the review questions/objectives lend themselves to a scoping review approach. | 2 |
| Objectives | 4 | Provide an explicit statement of the questions and objectives being addressed with reference to their key elements (e.g., population or participants, concepts, and context) or other relevant key elements used to conceptualize the review questions and/or objectives. | 3 |
| METHODS | | | |
| Protocol and registration | 5 | Indicate whether a review protocol exists; state if and where it can be accessed (e.g., a Web address); and if available, provide registration information, including the registration number. | NA |
| Eligibility criteria | 6 | Specify characteristics of the sources of evidence used as eligibility criteria (e.g., years considered, language, and publication status), and provide a rationale. | 4, NA |
| Information sources* | 7 | Describe all information sources in the search (e.g., databases with dates of coverage and contact with authors to identify additional sources), as well as the date the most recent search was executed. | 4 |
| Search | 8 | Present the full electronic search strategy for at least 1 database, including any limits used, such that it could be repeated. | 4 |
| Selection of sources of evidence† | 9 | State the process for selecting sources of evidence (i.e., screening and eligibility) included in the scoping review. | 4 |
| Data charting process‡ | 10 | Describe the methods of charting data from the included sources of evidence (e.g., calibrated forms or forms that have been tested by the team before their use, and whether data charting was done independently or in duplicate) and any processes for obtaining and confirming data from investigators. | 5 |
| Data items | 11 | List and define all variables for which data were sought and any assumptions and simplifications made. | 5 |
| Critical appraisal of individual sources of evidence§ | 12 | If done, provide a rationale for conducting a critical appraisal of included sources of evidence; describe the methods used and how this information was used in any data synthesis (if appropriate). | 5, Limitations section |
| Synthesis of results | 13 | Describe the methods of handling and summarizing the data that were charted. | 6 |
| RESULTS | | | |
| Selection of sources of evidence | 14 | Give numbers of sources of evidence screened, assessed for eligibility, and included in the review, with reasons for exclusions at each stage, ideally using a flow diagram. | 7 |
| Characteristics of sources of evidence | 15 | For each source of evidence, present characteristics for which data were charted and provide the citations. | 7 |
| Critical appraisal within sources of evidence | 16 | If done, present data on critical appraisal of included sources of evidence (see item 12). | NA, Limitations |
| Results of individual sources of evidence | 17 | For each included source of evidence, present the relevant data that were charted that relate to the review questions and objectives. | 9-20 |
| Synthesis of results | 18 | Summarize and/or present the charting results as they relate to the review questions and objectives. | 9-20 |
| DISCUSSION | | | |
| Summary of evidence | 19 | Summarize the main results (including an overview of concepts, themes, and types of evidence available), link to the review questions and objectives, and consider the relevance to key groups. | 21 |
| Limitations | 20 | Discuss the limitations of the scoping review process. | 26 |
| Conclusions | 21 | Provide a general interpretation of the results with respect to the review questions and objectives, as well as potential implications and/or next steps. | 26 |
| FUNDING | | | |
| Funding | 22 | Describe sources of funding for the included sources of evidence, as well as sources of funding for the scoping review. Describe the role of the funders of the scoping review. | 27 |

**Supplementary information, Table S1:** Table S2: Data extraction table on health security and PHC worldwide

| **Study** | **Country** | **Aim** | **Study** | **Issues** | **Concepts** | **Key Findings Related To Research Questions** |
| --- | --- | --- | --- | --- | --- | --- |
| Faudy et al 2011[1] | Indonesia | To assessing the preparedness of PHCs to response to potential disasters in their surrounding area. | short report | Disasters | Poor disaster preparedness | Shortage of quality of health staff, emergency facilities triage and fire management, transportation, lack of SOP and policy.  Need of promoting disaster preparedness, technical provision, including health staff training [1]. |
| Phalkey et al 2012[2] | India | to assess the functional capacity of the PHC system in Jagatsinghpur district of rural Orissa in India | qualitative | Disasters | Poor disaster preparedness in flood despite  prediction  Disaster preparedness plan | Poor prepared to of HF to handle the flood including electricity backup and essential medical supplies, HR along with missing SOP; pre-identified communication and incident command systems; effective leadership; and weak financial structures [2].  strengthening the response to future floods- developing facility specific preparedness plans with SOP during floods and identify clear lines of command [2] |
| Waynn and colleague 2012[3] | US | To evaluation of how collaboration between an Ontario public health unit and its p primary care providers facilitated an optimal response to influenza pandemic. | Commentary | Outbreaks | Integration of PHC and Public Health During a Public Health Emergency | Family health teams (integrated, interdisciplinary teams) provided flu assessment centers, with public health (e.g., providing infection control advice, funding, coordination, antiviral medication, clinical care guidelines, supplemental nurse staffing, and arrangement of communication strategies), offers a new capacity for timely, coordinated, and comprehensive response to public health emergencies, and provides a promising new direction for healthcare organization.[3] |
| Iwata et al 2012[4] | Japan | The team’s main goal was to detect the early signs of disease outbreaks. | Field report | Outbreaks | Post emergency outbreak  Post disaster surveillance | Challenges  Inadequate communication tools available, data collection team collected data in the forms, and summary findings reported daily to clinics. The team collaborated with public health nurse in rebuilding communication networks, alerted evacuation centres to epidemics. Modern health-care systems are highly vulnerable to the loss of advanced technological tools [4].  Mitigation.  The initiation or reestablishment – of disease surveillance following a natural disaster can prove challenging even in a developed country by (i) developing a surveillance system that is tailored to the local setting, (ii) establishing a support team network, and (iii) integrating the resources that remain – or soon become – locally available [4] |
| Gates 2015[5] | not specified | To discusses the lessons learned from the epidemic Ebola. | Perspective | Outbreaks | Next epidemic preparation -  reinforce basic public health systems  build a warning and response system for outbreaks. | Reinforce basic public health systems (e.g., PHC facilities, laboratories, surveillance systems, and critical care facilities, among other components). Ebola has spread faster and more widely in countries whose primary care systems were severely weakened by years of armed conflict and neglect.  lack of capacity for developing adaptable platforms, partly due to opportunity costs for private sector in shifting resources away from commercial projects to work on tools for future epidemics. An epidemic could set the world back drastically in the next few decades. [5].  Mitigation  Need of recruit local clinicians and train personnel rapidly into the affected countries, make diagnostic tests, drugs, and vaccine platforms.  Need of an international funding system that factors in opportunity costs. [5].building a global warning and response system-coordinated by a global institution that is given enough authority and funding to be effective;• enable fast decision making at a global level; expand investment in research and development and clarify regulatory pathways for developing new tools and approaches, improve early warning and detection systems ( scalable everyday systems and expandable during epidemic); involve a reserve corps of trained personnel and volunteers; strengthen health systems in LMICs; incorporate preparedness exercises in identification and improvement in the response system.[5] |
| Daw et al 2016[6] | Libya | assess the damage that affected Libyan health care system during the armed conflict | quantitative | Conflicts | The Libyan national health system is a victim of armed conflict | Impact- structural damage of PHCCs and hospitals, shortage of medical supplies (medical disposables and essential pharmaceuticals), lack of medical staff security, lack of communication and health care management).  Unusual problems emerged neglected orphans, dead bodies, and emergence of unusual infection in displaced provinces. [6] |
| stokke et al 2016[7] | not specified | To examines how research literature describes use of the PERS focusing on the users’ perspective | review | Disasters | Emergency Response System as a Technology Innovation in PHCS | users’ experiences and the consequences of having and using the alarm, and how the technology changes caring practices and interactions between the actors. [7]  The PERS contributes to safety and independent living for users of the alarm, well-established telecare technology in use interacts with the actors involved, creating changes in daily living and even affecting their identities.[7] |
| Moghadd et al 2017[8] | Eastern Mediterranean Region | As host to some of the world’s biggest emergencies and protracted crises, the Eastern Mediterranean Region carries the largest burden of displaced populations globally. | quantitative | Conflicts | Refugee needs, problems, and challenges  Health needs of IDPs | have shown generous hospitality towards displaced populations in the middle east region. Lebanon highest, followed by Jordan, Syrians, Iran hosts Afghan refugees, Pakistan has also IDPs and Afghan refugees. .[8]. With the majority of refugees and IDPs across the region living outside camp settings, both displaced populations and host communities, are exposed to increased public health risks including infectious diseases due to overcrowded living conditions, limited access to safe water and sanitation, and varying degrees of access to PHC services. .[8] |
| Attallah et al 2018[9] | not specified | To explore experience working on frontlines of social crises to understandings of pathways for equitable PHC. | qualitative | Conflicts | Developing Equitable PHC in Conflict-Affected Settings | Three themes emerged (a) Building Blocks, (b) Intermediating Factors, and (c) a Roadmap, which contributed conceptual frameworks explaining key contextually specific priorities, challenges, and facilitating factors for developing resilient health infrastructures under social crises. [9] |
| Raguin et al 2018[10] | central Africa | To move forward and begin to answer this question, we draw on lessons and experiences gained during the “global” health crises triggered by the HIV and Ebola pandemics. | commentary | Outbreaks | lessons from the HIV and Ebola epidemics.  nothing can be improved on a global or sustainable scale without | Need of a mandatory revolution in health governance—locally and globally and  re-examining the architecture and governance of major funding and international organizations dedicated to health[10]. Pressing economic, demographic, and climate issues related to health underscore the urgent need for these changes. PHC and community engagement, repositioning approaches to meet people’s needs, applying integrated disease management to respond to problems caused by the silo approach, implementing UHC, and ensuring equity are some of the new strategies [10]. |
| Shin et al 2018[11] | Sierra Leone | To examine the effectiveness of INGOs in a context of managing a fatal epidemic outbreak of Ebola in Sierra Leone; | Primary study | Outbreaks | INGOs Response Operations during Public Health Emergency | The development of healthcare infrastructures and provision of medical supplies have been significantly effective in terms of decreasing the severity of the crisis in chiefdoms.[11]. The policy tools which allow partners to enter in the field, can improve the effectiveness of INGOs’ responses in current and future epidemic outbreaks in LMICs.[11] |
| Assefa et al 2020[12] | Global | assess the relationship between GHS and UHC using two recent quantitative indices. | quantitative | Mixed | Global health security and UHC | There is a moderate and significant relationship between GHSI and UHCI and individual indices of UHCI. The risk of GHS threats a significant and negative correlation with the capacity for GHS and the capacity for UHC. The tension between GHS and UHC-global health agendas should be transformed into a synergistic solution. [12]. Strengthening the health systems, in tandem with the principles of PHC, and implementing a “One Health” approach will progressively enable countries to achieve both UHC and GHS towards a healthier and safer world [12] |
| Fuentes et al 2020[13] | Ecuador | To analyse the effects of the Pedernales earthquake on Ecuador’s health care system. | Mixed methods | Natural disasters | Effects of the Pedernales Earthquake on Ecuador’s Health Care System | Deficiencies in the health care system prior to the earthquake (the lack of doctors, nurses, and hospital beds). Poor preparedness health district for an emergency (e.g., earthquake), buildings fell and hospital disabled resulting in preventive action failures at the community level leading increase in diseases post-earthquake [13].  shortage of personnel and physical infrastructure, weaknesses in PHC, the lack of preparation, and limited availability of information on health indicators  sharp increase in pre-existing diseases in the area, and of new epidemic outbreaks[13] |
| Cohen et al 2020[14] | Israel | To examine the role of health-care services in the perceived community resilience of urban and suburban Arab communities in Israel in pandemic. | quantitative | Outbreaks | Health-Care Services as a Platform for Building Community Resilience among Minority Communities | Suburban community reported higher community resilience, more satisfied and confident in health-care services than residents of the urban community due to increased preparedness levels and strength of place attachment in the suburban community [14]. The satisfaction with primary health-care services, and not community type, significantly predicted community resilience. The pivotal role of PHC in building community resilience of minority communities in times of emergency and routine.[14] |
| David et al 2020[15] | Central African Republic | To understand this disparity between surveillance and public health, we examined selected moments in its history of surveillance and changing relations with public health structures | qualitative | Outbreaks | Hot spot – emergency and blind spot–PHC services lacking | Long-term continuities in privileging surveillance over the health system and population health, making the CAR a "hotspot" for emerging diseases and a "blind spot" of PHC. The country attracted considerable support for surveillance, without concomitant investment in public health system. Political disputes and financial constraints have obscured real primary care needs on the ground. [15]. As both a hotspot and a blind spot for global health, the CAR signals the need to reorient health interventions to address the long-term health of CAR people.[15] |
| Fitts et al 2020[16] | Australia | To highlight the vulnerabilities of remote ATSI communities in high prevalence of complex chronic disease and socio‐economic factors such as limited housing availability and overcrowding | commentary | Outbreaks | Remote health service vulnerabilities and responses to the COVID-19 pandemic | The capability of ATSIs and the ACCHS Sector, working with the government, to mitigate the threat of transmission rapidly and effectively in remote areas. Challenges- persistent workforce challenges faced by PHC services in remote, heavy reliance on short‐term or fly‐in, fly‐out/drive‐in, drive‐out staff, remote area nurses. [16]. easing of travel restrictions across the country brings the increased risk of transmission into remote areas. underscores the need to adequately plan and fund remote PHC services and ensure the availability of an adequate, appropriately trained local workforce.[16] |
| Santos et al 2020[17] | Brazil | To reiterate the importance of PHC | Qualitative | Outbreaks | PHC in Brazil during pandemic changes, challenges | a universal and free health system has existed and re-organized the PHC to attend the population. In pandemic, priority is given to the hospital sector and PHC systems is disrupted. Health system is being overloaded with the increase in covid cases. It is worth reflecting on the changes and challenges in PHC during pandemic.[17] |
| Juma et al 2020[18] | SSA | To explore the NCDs and COVID | commentaries | Outbreaks | A Wake-up Call for PHC System Strengthening in SSA for NCDs | investment is still insufficient in SSA while pandemic is a reminder that NCDs, which are increasingly prevalent in SSA, are closely interlinked to the burden of CD with exacerbating health outcomes. the pandemic in a sustainable and effective way to use the momentum created pivoting health spending towards PHC.[18] |
| Maciel at al [19] | Brazil | To discuss the reorganization of the CHWs work process because of the Covid-19 pandemic, considering its importance as a link between the community and the health services in the field of basic care. | review | Outbreaks | CHWs are the premise of PHC in the context of COVID-19 | The premises of PHC and on the axes of the CHW work in cultural competence and community orientation, aiming to discuss the changes introduced in this work regarding the following aspects: 1) health teams support, 2) use of telehealth, and 3) health education. [19]. The Covid-19 pandemic demanded reorganization of the work process and assistance flows in the field of basic care. For the CHW to continue developing their activities needs the guarantee decent working conditions, training and continuing education, including the concern about the possible discontinuity of other care needed to ensure the population health care in the territory.[19] |
| Miller et al 2020[20] | Yemen | To document the challenges to iCCM service delivery and to aid in developing strategies for overcoming service delivery bottlenecks in conflict-affected rural areas. | qualitative | Conflicts | ICCM in conflict affected setting | **Challenges -**The challenges in delivery of services were related to both a weak health system and the conflict. Policy, coordination, and funding challenges due to no integration of ICCM into the national health system but implemented as a short-term emergency program. CHW provide received services reduced access to services, especially during times of heightened conflict and insecurity, when CHWs could not travel. difficulties in travel due to the conflict challenged supervision, supply chain, and monitoring.  Travel was seen as the primary threat to the safety of CHWs and supervisors [20].Solution- use of mobile technology for supervision and data collection and pre-positioning of buffer stocks in locations closer to CHWs.  Risk reducing measure included travel during periods of heightened insecurity, safety training for CHWs, and use of mobile technology for communication. [20] |
| Omam et al 2020[21] | Cameroon | new normal beyond COVID-19 | qualitative | Outbreaks | In Cameroon, all stakeholders mobilised to respond to the outbreak | Little attention to strengthening the PHC system to contain the spread of the virus in communities, efforts are limited to central and regional laboratories and hospitals, forgetting the importance of PHC in emergency preparedness, response, and recovery.  WHO recommendation of involvement of PHC in triaging of patients presenting with COVID-19 symptoms while ensuring continuum of service[21]. |
| Prado et al 2020[22] | Many countries | To examines the PHC organization in response to the COVID-19 epidemic. | qualitative | Outbreaks | The international response of PHC to COVID-19 | different organizations and impacts of strategies to conduct actions according to the local characteristics of disease transmission, demography, public health services organization, and health system's capacity and financing, especially in the PHC area. In pandemic- increase in telephone and video consultations incorporating health information technology. An efficient PHC and essential actions, achieves more suitable results cumulative capacity or experience makes the difference facing the emerging demands on different health systems.[22] |
| Rai et al [23] | Indonesia | To develop the National action plan for health security began soon after the JEE, through multisectoral coordination and collaboration and with the support of a presidential instruction | Perspective | Mixed | Strengthening emergency preparedness and response systems | The development of the NAPHS with a focus on health system strengthening based on the PHC approach.  Innovative approach with inclusion of emergency preparedness in the mandatory minimum service standards for provincial and district governments. Articulate the importance of local emergency preparedness in Indonesia's decentralized governance through the development of contingency plans and simulation exercises for natural disasters and potential disease outbreaks.  Indonesia's extensive experience in pandemic influenza preparedness planning and exercises, integrated with a national disaster management system. Indonesia has signalled its commitment to implementing the NAPHS in full, focusing on enhanced emergency preparedness at all administrative levels.[23] |
| Rasanthan et al 2020[24] | not specified | To guide greater application of the PHC strategy, reflecting on tensions that remain between the political vision of PHC and its implementation in countries | policy and practice | Mixed | aspirations of PHC, arguing that national needs and action must dominate over global preoccupations. | Changing contexts and realities need to be accommodated. A clear distinction is needed between PHC as an inspirational vision and set of values for health development, and PHC as policy and implementation space. Stakeholders beyond the health sector will often need to lead, which is challenging because the concept of PHC is poorly understood by other sectors. Efforts on PHC as policy and implementation space might focus explicitly on primary care and the frontline of service delivery with clear links and support to complementary work on social determinants and building healthy societies.  Such efforts can be partial but important implementation solutions to contribute to the much bigger political vision of PHC.[24] |
| Rawaf et al 2020[25] | not specified | To summarise experiences of international primary care systems. | qualitative | Outbreaks | Lessons on the COVID-19 pandemic,  from primary care during the pandemic | During pandemic, primary care continued as the first point of contact to the health system but poorly informed policy makers on how to fulfil its role and ill equipped to provide care while protecting staff and patients against further spread of the infection.  The creativity and initiatives of local health professionals led to the introduction or extension of the use of telephone, e-mail, and virtual consulting, and introduced triaging to separate covid and non-covid case. Concerns of collateral damage to the health of the population due to abandoned or postponed routine care. The pandemic presents important lessons to strengthen health systems through better connection between public health, primary care, and secondary care to cope better with future waves of this and other pandemics.[25]. Covid-19 has had a complex impact on primary care, with improved access and coordination in many settings, balanced against resourcing and information flow issues, and a reduction in the comprehensiveness of services. it remains the cornerstone of pandemic response and has shown itself to be highly adaptable in meeting the unique demands of the pandemic and needs to be resourced, with sufficient equipment, training, and financing.[25] |
| Schmidt et al 2020[26] | US | To explore community response during a pandemic | qualitative | Outbreaks | Access to Care During a Pandemic: | Challenges- information gaps, supply shortages, and lack of resource coordination in caring for patients, which affected their ability to care for patients challenged care provided in the US. pre-emptive planning to prevent barriers to quality patient care, support disease surveillance and contact tracing, and optimize the use of limited resources (PPE, testing and hospital care). The experience of PHC providers during this pandemic provides useful information for understanding the gaps in emergency planning and management during a public health emergency.[26]  Improving Planning Efforts to Incorporate Community Primary Care Practices and Public Health Stakeholders [26] |
| Souza et al 2020[27] | Brazil | To explore SARS-CoV-2 and its association with severe pneumonia and deaths has exposed gaps in the health systems | short communication | Outbreaks | The need to strengthen PHC in Brazil in the context of the pandemic | PHC is the gateway to the health system plays a role in preventing, protecting, promoting, and treating individuals and communities. A universal and decentralized health system in Brazil has model of health re-organizing the health system. Reflection of the importance of strengthening PHC in Brazil in the times COVID-19 pandemic.[27] |
| Tappis et al 2020[28] | Yemen | To examines how RMNCAH+N services have been delivered since 2015 | qualitative | Outbreaks | Reproductive, maternal, newborn and child health service delivery | Efforts- humanitarian response in maintaining and functioning facilities, and deploying mobile clinics, outreach teams and CHVs networks to address urgent needs, attention specific child health services, and cholera control and treatment of acute malnutrition are given precedence over other services, HWs display notable resilience working in difficult conditions[28]. challenges resulting from insecurity, limited functionality of health facilities, and challenges in importation and distribution of supplies limit the availability and quality of services, lack of access due to insecurity, politicization of aid, weak health system capacity, costs of care seeking, and an ongoing cholera epidemic. [28] |
| Tumusiime et al 2020[29] | African region | To build health system resilience to facilitate service continuity during health threats, PHC revitalization, and health systems strengthening towards UHC. | meeting report | Mixed | Building health system resilience in the context of PHC revitalization for attainment of UHC: | Strategies for health system- working multisector ally/intersectorally, moving from fragmentation to integration, ensuring implementation and knowledge exchange, and rethinking resilience and embracing antifragility [29]. Deliberations from the Regional Forum are critical for the potential to directly inform policy and program design, given that the meeting convenes health sector technocrats, who are at the helm of policy design, action, and implementation.[29] |
| Baral 2021[30] | LMICs | To providing a synthesis of available evidence on the impact of pandemics and epidemics on (1) essential services and (2) health systems preparedness and strengthening. | review | Outbreaks | Health Systems and Services During pandemic | successful pandemic response depends on trust-of institutions, of science, and between communities and health systems. Previous lesson was inability of health systems to handle upcoming pandemics, lack of resources, infrastructure, and political, the reactive nature of policies and practices resulted in failed response. need for investments in implementation science and for strategies to bridge this persistent research-practice gap. Existing disparities in health systems and services being further exacerbated, with marginalized populations and LMICs burdened disproportionately. [30]. Need to further understand short- and long-term impacts of bypassed essential services, quality assurance of services, the role of PHC in the frontline, and the need for additional mechanisms for effective vaccine messaging and uptake during epidemics.[30]. |
| Dentico 2021[31] | not specified | To explore the global health governance has come to the fore at the intersection of the trajectories of global crises that have converged in 2020: | qualitative | Outbreaks | COVID-19 the soaring inequalities, the climate disaster, and the effects of a globalization | The COVID-19 imposes a new sense of purpose to health policymaking, which is not yet captured in the current failed global response to the pandemic.  An opportunity for the international community that believes in public health and the role of public institutions, to re-imagine itself and project new creative ways to engage beyond classical models, so as to reconquer some ground for a healthier future.[31] |
| Edelman et al 2021[32] | not specified | To summarize evidence from a diverse body of literature with a modification to accommodate four discrete phases of searching, screening, and eligibility assessment | review | Outbreaks | the enablers and barriers to implementing PHC in the pandemic | Enablers- investments in PHC improve equity and access, healthcare performance, accountability of health systems and health outcomes. Implementation of PHC enabled equity-informed financing models, health system and governance frameworks that differentiate multi-sectoral PHC from more discrete service-focussed primary care, and governance mechanisms that strengthen linkages between policymakers, civil society, non-governmental organizations, community-based organizations, and private sector entities.[32]. PHC implementation continues to grow, critical knowledge gaps are evident, particularly relating to country-level, context-specific governance, financing, workforce, accountability, and service coordination mechanisms. An agenda to guide future country specific PHC research is outlined.[32] |
| Fangfang et al 2021[33] | China | COVID-19 has affected PHC delivery in metropolitan areas. An integrated health-care system offers advantages in response to the community outbreak and transmission of highly infectious diseases | Perspective | Outbreaks | Integrated Healthcare Systems Response Strategies in pandemic | Integrated health-care system in the following effective strategies in response to the epidemic: (1) enhance the public workforce in primary health care; (2) integrate resources to allow regional sharing and efficient use; (3) employ teams centered on general practitioners for community containment; and (4) adopt e-health and telemedicine for healthcare delivery. [33].An integrated health-care system, core strategies and mechanisms can contribute to improving the public health capacity in emergency responses; they can transform health-care delivery in the COVID-19 epidemic. The experience in Shenzhen may help other cities in enhancing and coordinating the preparedness of their health-care systems in dealing with future public health emergency [33]. |
| Ghanizad et al 2021[34] | not specified | To discusses need for revitalisation of PHC as a powerful tool to save societies from pandemics' consequences. | letters to editor | Outbreaks | Revitalisation of PHC governance: an important pillar for the tangible management of COVID-19. | need for revitalisation of PHC as a powerful tool to save societies from pandemics' consequences.  impacts on social and economic activities confirm that attention to PHC is necessary for improved therapeutic resilience and tangible effects on the use of healthcare resources; and also mentions realisation of PHC governance as sustainable, productive, effective and fair needs global consensus.[34]. |
| Ibriahim et al 2021[35] | Iraq | To assess the resilience of health systems in four governorates affected by conflict from 2014 to 2018, and to convey recommendations. | qualitative | Conflicts | Resilience of health systems in conflict affected governorates of Iraq | (1) Absorption- The shock to the public sector health services by the ISIS invasion caught health services in the four governorates unprepared, with limited abilities to continue to provide services. Private pharmacies and private clinics in some places withstood the initial shock better than the public sector. (2) Adaptation- After the initial shock, many health facilities adapted by focusing on urgent needs for injury and communicable disease care. In most locations, MNCH preventive and promotive PHC services stopped. Ill persons would sometimes consult health workers in their houses at night for security reasons. (3) Restructuring or transformative activities- Some heavily damaged facilities are still functioning, but below precrisis level. Rebuilding lost community trust in the public sector is proving difficult. Health services had little preparation for and limited resilience to the ISIS influx. Governorates are still restructuring services after the liberation from ISIS in 2017.[35] |
| Kinder et al 2021[36] | not specified | To assess the degree PC and PH were integrated in national responses to the current coronavirus pandemic and learn. | short report | Outbreaks | Integrating primary care and public health to enhance response to a pandemic | PHC includes both PC and essential PH functions, the need to coordinate these two aspects, successful integration remains. A survey of stakeholders revealed many of the challenges encountered when facing the pandemic without a coordinated effort between PC and PH functions. uBy integrating PC and PH greater capacity to respond to emergencies may be possible if the synergies gained by harmonizing the two are realized.[36] |
| Bermejo et al 2021[37] | Cuba | to show how the Cuban National Health System has been able to ensure an effective and equitable response to COVID-19. | special report | Outbreaks | Equity and the Cuban National Health System's response to COVID-19 | in Cuba, a Plan for covid Prevention and Control was elaborated with multisectoral participation.  Preventive actions- preventive measures in the community, continued in the isolation centers and ended again in the community with actions of surveillance and follow up of recovered patients.  Treatment actions- molecular diagnosis laboratories were created in the province, free medical care, and treatment. Plan- the preparation of a single national intersectoral government plan; the use of strategies for research, diagnosis, and case tracing; and the implementation of a universal protocol for disease prevention and treatment of confirmed cases made it possible to control the disease with a health equity.[37] |
| Nayawad et al 2021[38] | Thailand | To explore the role of the health-care workforce to meet SDG targets, there still exists a shortage of professional health personnel in rural areas. | qualitative | Outbreaks | Community surveillance of COVID-19 by village health volunteers | The PHC system includes trained VHVs who provide basic health care to their communities. Relevant changes VHVs visited, identified, and monitored returnees and referred symptomatic patients to hospitals. The timely mobilization of Thailand’s trusted VHVs, educated and experienced in infectious disease surveillance, enabled the robust response to pandemic, contained without the use of a costly country-wide lockdown or widespread testing.[38] |
| Plagg et al 2021[39] | Italy | To identify challenges within PHC and hospital organization, cooperation between primary and specialist care, and access to health care services | qualitative | Outbreaks | PHC: Collapse of the First Line of Defence | Neglected PHC with a comparatively low availability of GPs per inhabitant, the initial prioritization of hospitals during the pandemic while neglecting PHC in terms of PPE, the lack of testing resources, and a failure to achieve coordinated support contributed to a quick overburdening of hospitals, where the dissolution of traditional departments into "macro-areas" may favour nosocomial infections in Lombardy. The flexible public healthcare services working in consistent cooperation with GPs, show better efficiency in containing viral spread and managing patients. Mitigation strategies-  Strengthening the PHC sector about human and technical resources and supporting the coordination between the different levels of HCPs help to avoid overcrowded hospitals, while protecting patients and HCPs during large-scale health emergencies.  in-depth analysis of structural determinants is needed in order to develop more-resilient and integrative health care systems.[39] |
| Ramadan et al 2021[40] | Cameroon, Democratic Republic of Congo, Mali, and Nigeria | To addresses the literature gap by applying a conflict intensity lens to the analysis of disparities in access to essential PHC services in four conflict-affected fragile states | quantitative | Conflicts | Access to primary healthcare Services in Conflict-Affected Fragile States | PHC access varied with more prevalent financial than geographic barriers to care. higher disparities both educational and wealth disparities with geographic proximity to medium or high intensity conflict. statistically significant interaction between conflict intensity and educational disparities in access to care. Both educational and wealth disparities in access to PHC services can be exacerbated by geographic proximity to organized violence. household surveys can contribute to healthcare assessment in conflict-affected and fragile settings.[40] |
| Ray and Mash 2021[41] | SSA | To synthesise the lessons learnt from the COVID-19 pandemic in the Africa region. | qualitative | Outbreaks | Innovation in PHC responses to COVID-19 | community-based activities; screening and testing; reorganisation of health services; emergency care; maintenance of essential non-COVID-19 health services; caring for the vulnerable; use of information technology; and reframing training opportunities. Vital role of CHWs for community resource, delivering medications to people of chronic conditions. Constraints - Difficulties with procurement of test kits and turn-around times, detriment of essential services and training of junior doctors. Strategies - use of internet technology for communication and remote consultations, contribution of family medicine principles, clear leadership and planning, multidisciplinary teamwork, and continuity of care.[41]. The community-orientated primary care approach, long-term benefits of technological innovations, and pandemic exposed the need to deliver on governmental commitments to strengthening PHC and UHC.[41] |
| Rispel et al 2021[42] | South Africa | To describe the health system response to the COVID-19 pandemic during the first epidemic wave in Gauteng province and to explore the perspectives of key informants on the provincial response. | qualitative | Outbreaks | Innovation, contestations, and fragilities of the health system response to COVID-19 | Gauteng developed an innovative, multi-sectoral and comprehensive provincial COVID-19 response to address challenge of saving lives and the economy. amplified the fragilities of existing systems, reflected in the corruption on PPE, poor data quality and inappropriate decisions on self-standing field hospitals, chronic under-investment and insufficient health workforce, the response failed to consider or deal with their fears, and to incorporate strategies for psychosocial support, and safe working environments, a de facto health system lockdown and reported collateral damage. missed opportunities to invest in PHC, partner with communities and to include the private health sector in the pandemic response. [42]  innovations of the multi-sectoral response to the COVID-19 pandemic, while addressing the contested areas and health system fragilities.[42] |
| Simen-Kapeu et al 2021[43] | Liberia | To review the community health policy development process to draw lessons from the health system strengthening efforts in Liberia post-EVD crisis. | qualitative | Outbreaks | Lessons learned from a health system approach to inform program design and better prepare for future shocks in Liberia | health system challenges and proposed policy and programmatic shifts to institutionalize a standardized CHP with fit for purpose and incentivized community health assistants to provide PHC services to the targeted populations. The CHP is in implementation and requires strengthened leadership, local capacities, and resources for sustainability. Lessons learned -the importance of establishing a coordination mechanism and leveraging partnership support; using a systems approach to better inform policy shifts; strengthening community engagement; and conducting evidence-based planning to inform policymakers. [43]. Community-based systems will play an even bigger role to toward building resilience for future shocks and strengthening PHC, which will require that communities be viewed as actors in the health system.[43] |
| Subba and Pradhan 2021[44] | not specified | To empower PHC institutions against COVID‑19 pandemic: A health system‑based approach | Editorial | Outbreaks | Empowering primary healthcare institutions against COVID‑19 pandemic: A health system‑based approach | PHC institutions are an essential foundation for the national response to COVID-19 disease in India. With the soaring number of confirmed cases, the health system is currently under unprecedented stress, there is a pressing need for empowering PHCIs in COVID-19 preparedness and response. The WHO "Health system building block" approach can work as a road map for the national health system in the process of empowering PHCIs and other upcoming emergencies.PHCIs institutions are going to play an indispensable role in the fight against COVID‑19. the current gaps in readiness will severely hamper the capacity of PHCIs to respond toward the pandemic over an extended period and reinstituted the importance of six building block approach toward health system[44]. it is imperative to adapt this at primary care level to bridge these gaps toward empowering PHCIs and future health system shocks. PHCIs would remain the fulcrum of the pandemic preparedness and response. our efforts towards moving from reactive to proactive approach with PHCIs.[44] |
| AbdulRahman et al 2021[45] | Dubai | To provide an insight on the feasibility and impact of telemedicine use among PHC providers and on nonurgent health care delivery in pandemic in UAE. | quantitative | Outbreaks | Digital Health Technology for Remote Care in Primary Care During the COVID-19 Pandemic: Experience from Dubai | There is 86% increased use in telemedicine service among adults, UAE nationals, female, and having government insurance, with general health consultation, covid related consultation, laboratory test requests. Virtual care services in PHC clinics have been highly successful in meeting the needs of patients during the COVID-19 pandemic. Telemedicine has enabled clinicians to continue providing care to patients while maintaining the necessary public health measures adopted in the fight against COVID-19 [45] |
| Caffery et al 2022[46] | Australia | To outlines how covid 19 operating environment has challenged traditional urban- dominated policy thinking about virtual health care delivery and how greater availability of telehealth appointments in remote Australia | commentary | Outbreaks | To access in‐person care expediated the introduction of virtual solutions in health service | Pandemic effect- distance is a decade- long problem for rural health access, the pandemic and associated restrictions on mobility have reduced in real terms the distance from, and time taken to critical services, unlocked health access for rural and remote areas due to lockdown, disrupted traditional delivery models and allowed the piloting of novel solutions, stress- testing current delivery systems, urban paternalism’ in understanding and delivering rural health.  Government policy changes to expand the MBS to include telephone or online health consultations are a positive initiative, the ongoing public health crisis, access parity for some rural and remote patients. telehealth has now become a permanent feature of the Medicare landscape, public health reforms for more flexible and inclusive UHC system but, needed steps towards improving access to PHC for patients in rural and remote areas.[46] |
| Downie et al 2022[47] | LMICs | To design a modular online training program for Remote Consulting in PHC (REaCH). | Mixed methods | Outbreaks | Remote Consulting in PHC in LMICs | Recommend the program to others, reported receiving relevant skills and applying their learning to their daily work, demonstrating satisfaction, learning, and perceived behavior change. identified several barriers to implementation of remote consulting, including lacking digital infrastructure, few resources, inflexible billing and record-keeping systems, and limited community awareness. The costs of data or airtime emerged as the greatest immediate barrier to supporting both the upscaling of REaCH training and subsequently the delivery of safe and trustworthy remote health care. The REaCH training program is feasible, acceptable, and effective in changing trainees' behavior [47]. |
| Gasparya et al 2022 [48] | not specified | to improve the accessibility of PHC and solve most health issues at this level. | review | Mixed | Global health is evolving aiming to explore needs and offering equitable health services | several global initiatives have been introduced to improve the accessibility of PHC and solve most health issues at this level. The 1978 Alma-Ata and 2018 Astana Declarations were the most important documents for a comprehensive approach to PHC services. the SDG in 2015, developments in all spheres of human life and multi-sectoral cooperation became the essential action targets that could contribute to improved health, well-being, and safety of all people. Riyadh Declaration on Digital Health and São Paulo Declaration on Planetary Health called to urgent action to employ advanced digital technologies, improve health data processing, and invest more in research management put to the test in the face of pandemic and other threats to humanity.[48] |
| Gonzalez et al 2022[49] | not specified | To maintain communication and supply routes is essential, as well as the guarantee of the autochthonous production of basic goods | review | Outbreaks | Lessons from COVID-19 for future disasters: | Pandemic highlighted to redefine the training plans for physicians to allow a more versatile and transversal training. Need of national health research plans to be able to respond quickly to questions posed by the various crises (use of resources, data and capabilities of the health sector), contingency plans( consider ethical aspects of patient needs), conflicts increase and require a bioethical response and respect for people's values; rapid, efficient and truthful communication systems in critic circumstances, the creation of National Coordination Centers for major disasters and Public Health can contribute to better face the crises of the future.[49] |
| Haggerty et al 2022[50] | US | To understand how telemedicine delivery of family medicine care affects patient access and visit completion rates in a rural primary care setting. | quantitative | Mixed | Impact on Visit Completion Rate in a Rural Appalachian Population | Telemedicine can increase completion rates by about 20% among, working-aged persons are more likely to complete telemedicine visits, older persons with higher risk scores are more likely to complete their visits if they use telemedicine. [50]. Telemedicine can be a tool to improve patient access to primary care in rural populations, telemedicine may facilitate access to care for difficult-to-reach patients ( in rural areas, rigid work schedules, live longer distances from the clinic, complex health problems, and poor and lower education).[50] |
| Jiaviriyaboonya 2022[51] | Thailand | To pay attention to the roles and functions of VHVs, as well as to the process of social network formation of the VHVs in Nakhon Phanom. | qualitative | Outbreaks | VHVs socio-political network in minimizing risk and managing the crisis during COVID-19 | Covid response in Thailand- mobilizing a variety of local resources, and such mobilization is operated and maintained by numerous local agencies or relevant stakeholders, the VHVs formulate "socio-political networks," or can be seen as a "pluralistic network" based on a "collaborative system" between numerous agents/stakeholders in the community, including VHV groups, villagers, families/households, local politicians/officials, and private sector actors. fundamental research applying to understand the larger societies where community collaborations, social networks, and social capital are key mechanisms empowering agencies to encounter the invasion of a global pandemic.[51] |
| Karamagi, et al 2022[52] | multicounty | To present short summaries and experiences and their practices and outcomes. | qualitative | Mixed | Cross country lessons sharing on practices, challenges in PHC | Strategies (i) defining and making more essential health services available, (ii) increasing service coverage targeting hard to reach populations, (iii) financial risk protection, (iv) improving user satisfaction with services, (v) improving health security, and (vi) improving coverage with health related sector services.[52] |
| Kumpunen et al 2022[53] | Europe | To develop an analysis framework examining the models of PHC delivery employed by PHC providers in response to the pandemic. | qualitative | Outbreaks | Transformations in the landscape of PHC in pandemic | Model of delivery (1) multi-disciplinary primary care teams coordinating with public health to deliver the emergency response and essential services; (2) PHC providers defining and identifying vulnerable populations for medical and social outreach; and (3) PHC providers employing digital solutions for remote triage, consultation, monitoring and prescriptions to avoid unnecessary contact. [53]. |
| Lal et al 2022[54] | multicounty | To Minimizing COVID-19 disruption:  assessing the unique and timely steps the Strategic Fund took to support the COVID-19 response | review | Outbreaks | Ensuring the supply of essential health products for health emergencies and routine health services | Leverage resources through multi-country pooled procurement enables countries to increase access to quality affordable essential medicines and supplies that meet priority health objectives and respond to health emergencies. Strategies (Strategic partnerships and tools for supply chain disruptions and streamline procurement and deployment, mitigating stockouts and ensuring cost efficiencies across various therapeutic areas). optimize usage of pooled procurement mechanisms facilitated, multilateral technical cooperation and other regional mechanisms (PAHO fund), help prepare for future health crises while maintaining essential health services.[54] |
| leao et al 2022[55] | Portugal | to inform how individual and institutional factors contributed for the preparedness to respond during the first months of a public health emergency. | qualitative | Outbreaks | determinants of willingness and readiness to respond in the onset of pandemic | Determinants- perception of adequate infrastructures, lack of access to personal protective equipment and organization, the perception of not being able to make a difference, risk of work-related burnout and experiencing colleagues or patients' deaths due to COVID-19. Adequate organization, infrastructures, and access to PPE for workers' preparedness in a new public health emergency, as well workers' understanding of their roles and expected impact, the planning of the response of healthcare institutions in future public health emergencies.[55] |
| Mason et al 2022[56] | LMICs | To inform the development of digital health tools to support public health objectives such as the Sustainable Development Goals. | qualitative | Outbreaks | Lessons Learned from Implementing Digital Health Tools to Address COVID-19 in LMICs | Lesson learned (1) user-centered design is key to the widespread adoption of digital tools; (2) strong, country-led partnerships are essential for scaling up and sustaining digital tools; and (3) using adaptable digital tools enables implementers to focus on the content of the solution rather than the technology.  Lessons learned from implementing and adapting digital tools for additional health applications (bolstering PHC, reaching vulnerable and marginalized populations, and empowering health workers with the real-time information necessary to optimize their work and improve the health), focus on robust monitoring and evaluation of digital tools and sustainable financing models.[56] |
| Peterson et al 2022[57] | not specified | to understanding UHC and PHC is evaluated in the light of the influence of political economy on health systems | special report | Outbreaks | UHC and political economy, neoliberalism, and effects of pandemic | Sturmberg and Martin's application of systems and complexity theory to understanding UHC and PHC is evaluated in the light of the influence of political economy on health systems. the role that neoliberal approaches to governance have had in creating increased inequities is seen as a key challenge for UHC. COVID-19 has emphasized long standing discrepancies in health and these disadvantages require government will and cooperation together with adequate social services to redress these discrepancies in UHC.[57] |
| Phiri et al 2022[58] | Malawi | To mitigate against COVID-19 are the primary healthcare facilities, strategically placed throughout districts to offer primary and maternal healthcare. | Mixed methods | Outbreaks | Preparedness for and impact of COVID-19 on PHC delivery in urban and rural Malawi | Most frontline HWs received training and access to preventative COVID-19 material, disruptions to key services and a reduction in clients attending facilities. Key barriers included periodic shortages of resources (soap, hand sanitiser, water, masks and staff), managing physical distancing and in handling suspected cases, discrepancies between reported behaviour and practice, particularly with consistent use of masks, negatively impacted their lives, experienced fatigue and stress due to heavy workloads, stigma in the community and worries about becoming infected, resource (human and material) inadequacy shaped the HF capacity for support and response to COVID-19, and frontline workers may require psychosocial support to manage the impacts of the COVID-19 pandemic.[58] |
| Silva et al 2022[59] | Global | To map the use of digital health strategies in PHC worldwide and their impact on quality of care during the COVID-19 pandemic | review | Outbreaks | Digital Health Opportunities to Improve PHC in the Context of COVID-19: | Nomenclatures of digital strategies adopted; types of information and communication technologies; characteristics of digital strategies in PHC; impacts on quality of care; and benefits, limitations, and challenges of digital strategies in PHC. The impacts on organization of quality of care, demonstrating the strengthening of (1) continuity of care; (2) economic, social, geographical, time, and cultural accessibility; (3) coordination of care; (4) access; (5) integrality of care; (6) optimization of appointment time; (7) and efficiency. [59]. Negative impacts (reduced access to services and increased inequity and unequal use of services offered, digital exclusion of part of the population, lack of planning for defining the role of professionals, disarticulation of actions with real needs of the population, fragile articulation between remote and face-to-face modalities, and unpreparedness of professionals to meet demands using digital technologies) .[59]. |
| Stengel et al 2022[60] | Germany | to explore the responses of PCP during the early COVID‑19 pandemic and to analyze these with a view on the resilience of the PHC system from the PCPs. | qualitative | Outbreaks | Resilience of the PHC system – German primary care practitioners’ perspectives during the early pandemic | Primary care had an overall strong ability to adapt and show resilience, albeit with wide variance in speed and scope of the responses. When coping with uncertainty, the reasons given by PCPs in favour of opening a CCP involved intrinsic motivation and self‑initiative (the lack of PPE, problems with space, and worries about organizational burden), a strong association existed between the establishment of a CCP and the use of resources (i.e., existing networks, personal protective equipment, exercising an office of professional political function). [60] |
| Tan et al 2022[61] | China | To focuses on the role of primary care in China's response to COVID-19. | qualitative | Outbreaks | Reflections on China's primary care response to COVID-19: roles, limitations, and implications | At the peak of the pandemic, primary care providers shoulder various public health responsibilities and work in close partnerships with other key stakeholders in the local communities. Primary care providers keep playing a 'sentinel'/surveillance role in identifying re-emerging cases after the elimination of community transmissions of COVID-19. the pandemic once again highlights some key limitations of the primary care sector, including the lack of gatekeeping, limited capacity and weak integration between medical care and public health.[61] |
| Taylor et al 2022[62] | New Zee Land | To learn from PHC experts' experiences from the COVID-19 pandemic across countries. | qualitative | Outbreaks | Multinational PHC experiences from the initial wave of the COVID-19 pandemic | PHC response directly influenced by the pandemic: 1) impact on the primary care workforce (task-shifting responsibilities outside clinician specialty and changes in scope of work, financial strains on practices, and the daily uncertainties and stress of a constantly evolving situation), 2) impact on patient care delivery, both essential care for COVID-19 cases and the non-essential care that was neglected or postponed; 3) and the shift to using new technologies. PHC experiences with the COVID-19 pandemic across the globe were similar in their levels of workforce stress, rapid technologic adaptation, and need to pivot delivery strategies, often at the expense of routine care.[62] |
| Vivalya et al 2022[63] | Congo | To review of available literature regarding mental illness in armed conflict and EVD outbreak settings. | Review | Outbreaks | Developing mental health services during and in the aftermath of the Ebola virus disease outbreak in armed conflict settings | The burden of mental illness is consistent, but mental healthcare is not integrated into PHC, requires the involvement of affected communities in their problem-solving process. The implementation of a comprehensive mental health care, through the application of mental health Gap Action Program (mhGAP) at community level and calls for further implementation research perspectives on the integration of mental healthcare into the health system of areas affecting by civil instability and natural disasters. poor implementation of community mental health services into PHC in regions affected by armed conflict and natural disasters. Stakeholders need to rethink to implementation of mhGAP into the emergency response against outbreaks and natural disasters.[63] |
| Xu et al 2022[64] | China | To analyse COVID-19 influences on the design, implementation, and validity of assessing the quality of PHC using unannounced standardized patients (USPs) | qualitative | Outbreaks | Covid outbreaks and management | Our experiences suggest that the pandemic created not only barriers but also opportunities to innovate ways to build a resilient data collection system. To build data system reliance, we recommend harnessing the power of technology for a hybrid model of remote and in-person work, learning from the sharing economy to pool strengths and optimize resources, and dedicating individual and group leadership to problem-solving and results[64] |

# References

1. Fuady A, Pakasi TA, Mansyur M: **Primary Health Centre disaster preparedness after the earthquake in Padang Pariaman, West Sumatra, Indonesia**. *BMC Res Notes* 2011, **4**:81.

2. Phalkey R, Runge-Ranzinger S, Marx M, Dash SR, Mukhopadhyay A: **Prepared to react? Assessing the functional capacity of the primary health care system in rural Orissa, India to respond to the devastating flood of September 2008**. *Global Health Action* 2012, **5**:1-10.

3. Wynn A, Moore KM: **Integration of primary health care and public health during a public health emergency**. *Am J Public Health* 2012, **102**(11):e9-e12.

4. Iwata O, Oki T, Ishiki A, Shimanuki M, Fuchimukai T, Chosa T, Shoichi C, Nakamura Y, Shima H, Kanno M *et al*: **Infection surveillance after a natural disaster: Lessons learnt from the Great East Japan Earthquake of 2011**. *Bulletin of the World Health Organization* 2013, **91**(10):784-789.

5. Gates B: **The next epidemic: Lessons from Ebola**. *The New England Journal of Medicine* 2015, **372**(15):1381-1384.

6. Daw MA, El-Bouzedi A, Dau AA: **The assessment of efficiency and coordination within the Libyan health care system during the armed conflict-2011**. *Clinical Epidemiology and Global Health* 2016, **4**(3):120-127.

7. Stokke R: **The Personal Emergency Response System as a Technology Innovation in Primary Health Care Services: An Integrative Review**. *J Med Internet Res* 2016, **18**(7):e187.

8. Moghaddam HT, Sayedi SJ, Moghadam ZE, Bahreini A, Abbasi MA, Saeidi M: **Refugees in the Eastern Mediterranean Region: Needs, Problems and Challenges**. *International Journal of Pediatrics-Mashhad* 2017, **5**(3):4625-4639.

9. Atallah DG, Djalali A, Fredricks K, Arlington L, Bussio M, Nelson BD: **Developing Equitable Primary Health Care in Conflict-Affected Settings: Expert Perspectives From the Frontlines**. *Qual Health Res* 2018, **28**(1):98-111.

10. Raguin G, Girard PM: **Toward a global health approach: lessons from the HIV and Ebola epidemics**. *Global Health* 2018, **14**(1):114.

11. Shin YA, Yeo J, Jung K: **The Effectiveness of International Non-Governmental Organizations' Response Operations during Public Health Emergency: Lessons Learned from the 2014 Ebola Outbreak in Sierra Leone**. *Int J Environ Res Public Health* 2018, **15**(4).

12. Assefa Y, Hill PS, Gilks CF, Van Damme W, van de Pas R, Woldeyohannes S, Reid S: **Global health security and universal health coverage: Understanding convergences and divergences for a synergistic response**. *Plos One* 2020, **15**(12).

13. Cañizares Fuentes R, Barquet Abi Hanna G, Santana Véliz C, Blasco Carlos M: **Effects of the Pedernales Earthquake on Ecuador's Health Care System**. *Disaster Medicine and Public Health Preparedness* 2020.

14. Cohen O, Mahagna A, Shamia A, Slobodin O: **Health-Care Services as a Platform for Building Community Resilience among Minority Communities: An Israeli Pilot Study during the COVID-19 Outbreak**. *Int J Environ Res Public Health* 2020, **17**(20).

15. David PM, Nakouné E, Giles-Vernick T: **Hotspot or blind spot? Historical perspectives on surveillance and response to epidemics in the Central African Republic**. *Int J Public Health* 2020, **65**(3):241-248.

16. Fitts MS, Russell D, Mathew S, Liddle Z, Mulholland E, Comerford C, Wakerman J: **Remote health service vulnerabilities and responses to the COVID‐19 pandemic**. *Australian Journal of Rural Health* 2020, **28**(6):613-617.

17. Gois-Santos VT, Santos VS, Souza CDF, Tavares CSS, Gurgel RQ, Martins-Filho PR: **Primary Health Care in Brasil in the times of COVID-19: changes, challenges and perspectives**. *Rev Assoc Med Bras (1992)* 2020, **66**(7):876-879.

18. Kraef C, Juma P, Kallestrup P, Mucumbitsi J, Ramaiya K, Yonga G: **The COVID-19 Pandemic and Non-communicable Diseases—A Wake-up Call for Primary Health Care System Strengthening in Sub-Saharan Africa**. *Journal of Primary Care & Community Health* 2020, **11**:1-3.

19. Maciel FBM, Santos H, Carneiro R, Souza EA, Prado N, Teixeira CFS: **Community health workers: reflections on the health work process in Covid-19 pandemic times**. *Cien Saude Colet* 2020, **25**(suppl 2):4185-4195.

20. Miller NP, Zunong N, Al-Sorouri TAA, Alqadasi YM, Ashraf S, Siameja C: **Implementing integrated community case management during conflict in Yemen**. *J Glob Health* 2020, **10**(2):020601.

21. Omam Ngo Bibaa L-A: **Primary health care beyond COVID-19: dealing with the pandemic in Cameroon**. *BJGP Open* 2020, **4**(4):1-4.

22. Prado N, Rossi TRA, Chaves SCL, de Barros SG, Magno L, dos Santos H, dos Santos AM: **The international response of primary health care to COVID-19: document analysis in selected countries**. *Cadernos De Saude Publica* 2020, **36**(12).

23. Rai NK, Rim KI, Wulandari EW, Subrata F, Sugihantono A, Sitohang V: **Strengthening emergency preparedness and response systems: experience from Indonesia**. *WHO South East Asia J Public Health* 2020, **9**(1):26-31.

24. Rasanathan K, Evans TG: **Primary health care, the Declaration of Astana and COVID-19**. *Bull World Health Organ* 2020, **98**(11):801-808.

25. Rawaf S, Allen LN, Stigler FL, Kringos D, Yamamoto HQ, van Weel C, Global Forum Universal Hlth C: **Lessons on the COVID-19 pandemic, for and by primary care professionals worldwide**. *European Journal of General Practice* 2020, **26**(1):129-133.

26. Schmidt ME, von Fricken ME, Wofford RN, Libby RC, Maddox PJ: **Access to Care During a Pandemic: Improving Planning Efforts to Incorporate Community Primary Care Practices and Public Health Stakeholders**. *World Medical & Health Policy* 2020, **12**(3):274-281.

27. Souza CDF, Gois-Santos VT, Correia DS, Martins-Filho PR, Santos VS: **The need to strengthen Primary Health Care in Brazil in the context of the COVID-19 pandemic**. *Braz Oral Res* 2020, **34**:e047.

28. Tappis H, Elaraby S, Elnakib S, AlShawafi NAA, BaSaleem H, Al-Gawfi IAS, Othman F, Shafique F, Al-Kubati E, Rafique N *et al*: **Reproductive, maternal, newborn and child health service delivery during conflict in Yemen: a case study**. *Confl Health* 2020, **14**:30.

29. Tumusiime P, Karamagi H, Titi-Ofei R, Amri M, Seydi ABW, Kipruto H, Droti B, Zombre S, Yoti Z, Zawaira F *et al*: **Building health system resilience in the context of primary health care revitalization for attainment of UHC: proceedings from the Fifth Health Sector Directors' Policy and Planning Meeting for the WHO African Region**. *BMC Proc* 2020, **14**(Suppl 19):16.

30. Baral P: **Health Systems and Services During COVID-19: Lessons and Evidence From Previous Crises: A Rapid Scoping Review to Inform the United Nations Research Roadmap for the COVID-19 Recovery**. *Int J Health Serv* 2021, **51**(4):474-493.

31. Dentico N: **The Breathing Catastrophe: COVID-19 and Global Health Governance**. *Development (Rome)* 2021, **64**(1-2):4-12.

32. Edelman A, Marten R, Montenegro H, Sheikh K, Barkley S, Ghaffar A, Dalil S, Topp SM: **Modified scoping review of the enablers and barriers to implementing primary health care in the COVID-19 context**. *Health Policy Plan* 2021, **36**(7):1163-1186.

33. Fangfang G, Guangyu HU, Hanqun LIN, Xizhuo SUN, Wenxin W: **Integrated Healthcare Systems Response Strategies Based on the Luohu Model During the COVID-19 Epidemic in Shenzhen, China**. *International Journal of Integrated Care (IJIC)* 2021, **21**(1):1-7.

34. Ghanizadeh G, Masoumbeigi H, Hosseini-Shokouh S-M: **Revitalisation of primary health care governance: an important pillar for the tangible management of COVID-19**. In*.*, vol. 13. Clayton, VIC, <Blank>: CSIRO Publishing; 2021: 313-314.

35. Ibrahim S, Al-Dahir S, Al Mulla T, Lami F, Hossain SMM, Baqui A, Burnham G: **Resilience of health systems in conflict affected governorates of Iraq, 2014–2018**. *Conflict and Health* 2021, **15**(1).

36. Kinder K, Bazemore A, Taylor M, Mannie C, Strydom S, George J, Goodyear-Smith F: **Integrating primary care and public health to enhance response to a pandemic**. *Prim Health Care Res Dev* 2021, **22**:e27.

37. Mas Bermejo P, Sánchez Valdés L, Somarriba López L, Valdivia Onega NC, Vidal Ledo MJ, Alfonso Sánchez I, Seuc Jo A, Almeida Cruz Y, Morales Ojeda R: **Equity and the Cuban National Health System's response to COVID-19**. *Rev Panam Salud Publica* 2021, **45**:e80.

38. Nayawadee K, Ratrawee P, Nithikorn S, Shinji N: **Community surveillance of COVID-19 by village health volunteers, Thailand**. *Bulletin of the World Health Organization* 2021, **99**(5):393-397.

39. Plagg B, Piccoliori G, Oschmann J, Engl A, Eisendle K: **Primary Health Care and Hospital Management During COVID-19: Lessons from Lombardy**. *Risk Manag Healthc Policy* 2021, **14**:3987-3992.

40. Ramadan M, Tappis H, Uribe MV, Brieger W: **Access to primary healthcare Services in Conflict-Affected Fragile States: a subnational descriptive analysis of educational and wealth disparities in Cameroon, Democratic Republic of Congo, Mali, and Nigeria**. *Int J Equity Health* 2021, **20**(1):253.

41. Ray S, Mash R: **Innovation in primary health care responses to COVID-19 in Sub-Saharan Africa**. *Prim Health Care Res Dev* 2021, **22**:e44.

42. Rispel LC, Marshall C, Matiwane B, Tenza IS: **Innovations, contestations and fragilities of the health system response to COVID-19 in the Gauteng Province of South Africa**. *PLoS One* 2021, **16**(12):e0261339.

43. Simen-Kapeu A, Lewycka S, Ibe O, Yeakpalah A, Horace JM, Ehounou G, Boima T, Wesseh CS: **Strengthening the community health program in Liberia: Lessons learned from a health system approach to inform program design and better prepare for future shocks**. *J Glob Health* 2021, **11**:07002.

44. Subba SH, Pradhan SK, Sahoo BK: **Empowering primary healthcare institutions against COVID-19 pandemic: A health system-based approach**. *J Family Med Prim Care* 2021, **10**(2):589-594.

45. AbdulRahman M, Al-Tahri F, AlMehairi MK, Carrick FR, Aldallal AMR: **Digital Health Technology for Remote Care in Primary Care During the COVID-19 Pandemic: Experience from Dubai**. *Telemed J E Health* 2022, **28**(8):1100-1108.

46. Caffery LA, Muurlink OT, Taylor‐Robinson AW: **Survival of rural telehealth services post‐pandemic in Australia: A call to retain the gains in the 'new normal'**. *Australian Journal of Rural Health* 2022, **30**(4):544-549.

47. Downie A, Mashanya T, Chipwaza B, Griffiths F, Harris B, Kalolo A, Ndegese S, Sturt J, De Valliere N, Pemba S: **Remote Consulting in Primary Health Care in Low- and Middle-Income Countries: Feasibility Study of an Online Training Program to Support Care Delivery During the COVID-19 Pandemic**. *JMIR Form Res* 2022, **6**(6):e32964.

48. Gasparyan AY, Kumar AB, Yessirkepov M, Zimba O, Nurmashev B, Kitas GD: **Global Health Strategies in the Face of the COVID-19 Pandemic and Other Unprecedented Threats**. *J Korean Med Sci* 2022, **37**(22):e174.

49. González Del Castillo J, Martín-Delgado MC, Martín Sánchez FJ, Martínez-Sellés M, Molero García JM, Moreno Guillén S, Rodríguez-Artalejo FJ, Ruiz-Galiana J, Cantón R, De Lucas Ramos P *et al*: **Lessons from COVID-19 for future disasters: an opinion paper**. *Rev Esp Quimioter* 2022.

50. Haggerty T, Stephens HM, Peckens SA, Bodkins E, Cary M, Dino GA, Sedney CL: **Telemedicine versus in-Person Primary Care: Impact on Visit Completion Rate in a Rural Appalachian Population**. *J Am Board Fam Med* 2022, **35**(3):475-484.

51. Jiaviriyaboonya P: **Anthropological study of village health volunteers' (VHVs') socio-political network in minimizing risk and managing the crisis during COVID-19**. *Heliyon* 2022, **8**(1):e08654.

52. Karamagi H, Titi-Ofei R, Amri M, Zombre S, Kipruto H, Seydi ABW, Avortri G, Nabyonga J, Tumusiime P: **Cross country lessons sharing on practices, challenges and innovation in primary health care revitalization and universal health coverage implementation among 18 countries in the WHO African Region**. *Pan African Medical Journal* 2022, **41**.

53. Kumpunen S, Webb E, Permanand G, Zheleznyakov E, Edwards N, van Ginneken E, Jakab M: **Transformations in the landscape of primary health care during COVID-19: Themes from the European region**. *Health Policy* 2022, **126**(5):391-397.

54. Lal A, Lim C, Almeida G, Fitzgerald J: **Minimizing COVID-19 disruption: Ensuring the supply of essential health products for health emergencies and routine health services**. *Lancet Reg Health Am* 2022, **6**:100129.

55. Leão T, Duarte G, Gonçalves G: **Preparedness in a public health emergency: determinants of willingness and readiness to respond in the onset of the COVID-19 pandemic**. *Public Health* 2022, **203**:43-46.

56. Mason C, Lazenby S, Stuhldreher R, Kimball M, Bartlein R: **Lessons Learned From Implementing Digital Health Tools to Address COVID-19 in LMICs**. *Front Public Health* 2022, **10**:859941.

57. Peterson CL, Walker C: **Universal health care and political economy, neoliberalism and effects of COVID-19: A view of systems and complexity**. *J Eval Clin Pract* 2022, **28**(2):338-340.

58. Phiri MM, MacPherson EE, Panulo M, Chidziwisano K, Kalua K, Chirambo CM, Kawalazira G, Gundah Z, Chunda P, Morse T: **Preparedness for and impact of COVID-19 on primary health care delivery in urban and rural Malawi: a mixed methods study**. *BMJ Open* 2022, **12**(6):e051125.

59. Silva C, Lopes RH, de Goes Bay O, Jr., Martiniano CS, Fuentealba-Torres M, Arcêncio RA, Lapão LV, Dias S, Uchoa S: **Digital Health Opportunities to Improve Primary Health Care in the Context of COVID-19: Scoping Review**. *JMIR Hum Factors* 2022, **9**(2):e35380.

60. Stengel S, Roth C, Breckner A, Cordes L, Weber S, Ullrich C, Peters-Klimm F, Wensing M: **Resilience of the primary health care system – German primary care practitioners’ perspectives during the early COVID-19 pandemic**. *BMC Primary Care* 2022, **23**(1).

61. Tan X, Liu C, Wu H: **Reflections on China's primary care response to COVID-19: roles, limitations and implications**. *Prim Health Care Res Dev* 2022, **23**:e46.

62. Taylor MK, Kinder K, George J, Bazemore A, Mannie C, Phillips R, Strydom S, Goodyear-Smith F: **Multinational primary health care experiences from the initial wave of the COVID-19 pandemic: A qualitative analysis**. *SSM Qual Res Health* 2022, **2**:100041.

63. Vivalya BMN, Vagheni MM, Kitoko GMB, Vutegha JM, Kalume AK, Piripiri AL, Masika YD, Mbeva JK: **Developing mental health services during and in the aftermath of the Ebola virus disease outbreak in armed conflict settings: a scoping review**. *Global Health* 2022, **18**(1):71.

64. Xu DR, Cai Y, Wang X, Chen Y, Gong W, Liao J, Zhou J, Zhou Z, Zhang N, Tang C *et al*: **Improving Data Surveillance Resilience Beyond COVID-19: Experiences of Primary heAlth Care quAlity Cohort In ChinA (ACACIA) Using Unannounced Standardized Patients**. *Am J Public Health* 2022, **112**(6):913-922.
